# Transcriptome analysis of MENX-associated rat pituitary adenomas identifies novel molecular mechanisms involved in the pathogenesis of human pituitary gonadotroph adenomas

**DOI:** 10.1007/s00401-013-1132-7

**Published:** 2013-06-12

**Authors:** Misu Lee, Ilaria Marinoni, Martin Irmler, Tsambika Psaras, Jürgen B. Honegger, Rudi Beschorner, Natasa Anastasov, Johannes Beckers, Marily Theodoropoulou, Federico Roncaroli, Natalia S. Pellegata

**Affiliations:** 1 Institute of Pathology, Helmholtz Zentrum München, Neuherberg, Germany; 2 Institute of Experimental Genetics, Helmholtz Zentrum München, Neuherberg, Germany; 3 Department of Neurosurgery, University of Tübingen, Tübingen, Germany; 4 Department for Neuropathology, Institute for Pathology and Neuropathology, University of Tübingen, Tübingen, Germany; 5 Institute of Radiation Biology, Helmholtz Zentrum München, Neuherberg, Germany; 6 Technical University Munich, Chair of Experimental Genetics, Am Hochanger 8, 85350 Freising-Weihenstephan, Germany; 7 Max-Planck Institute for Psychiatry, Munich, Germany; 8 “John Fulcher” Neuro-Oncology Lab, Imperial College, London, UK; 9 Present Address: Institute of Pathology, University of Bern, Bern, Switzerland

**Keywords:** Pituitary gonadotroph adenoma, Transcriptome analysis, MENX model, CYP11A1, NUSAP1

## Abstract

Gonadotroph adenomas comprise 15–40 % of all pituitary tumors, are usually non-functioning and are often large and invasive at presentation. Surgery is the first-choice treatment, but complete resection is not always achieved, leading to high recurrence rates. As gonadotroph adenomas poorly respond to conventional pharmacological therapies, novel treatment strategies are needed. Their identification has been hampered by our incomplete understanding of the molecular pathogenesis of these tumors. Recently, we demonstrated that MENX-affected rats develop gonadotroph adenomas closely resembling their human counterparts. To discover new genes/pathways involved in gonadotroph cells tumorigenesis, we performed transcriptome profiling of rat tumors versus normal pituitary. Adenomas showed overrepresentation of genes involved in cell cycle, development, cell differentiation/proliferation, and lipid metabolism. Bioinformatic analysis identified downstream targets of the transcription factor SF-1 as being up-regulated in rat (and human) adenomas. Meta-analyses demonstrated remarkable similarities between gonadotroph adenomas in rats and humans, and highlighted common dysregulated genes, several of which were not previously implicated in pituitary tumorigenesis. Two such genes, *CYP11A1* and *NUSAP1*, were analyzed in 39 human gonadotroph adenomas by qRT-PCR and found to be up-regulated in 77 and 95 % of cases, respectively. Immunohistochemistry detected high P450scc (encoded by *CYP11A1*) and NuSAP expression in 18 human gonadotroph tumors. In vitro studies demonstrated for the first time that *Cyp11a1* is a target of SF-1 in gonadotroph cells and promotes proliferation/survival of rat pituitary adenoma primary cells and cell lines. Our studies reveal clues about the molecular mechanisms driving rat and human gonadotroph adenomas development, and may help identify previously unexplored biomarkers for clinical use.

**Electronic supplementary material:**

The online version of this article (doi:10.1007/s00401-013-1132-7) contains supplementary material, which is available to authorized users.

## Introduction

Clinically non-functioning adenomas of the pituitary gland account for about 30 % of all adenomas. About 80 % of them belong to the gonadotroph lineage. They are usually diagnosed when signs and symptoms of mass effects occur, and about 40 % of them extend to the cavernous sinus and, less commonly, invade the sellar floor, making their resection and post-operative radiotherapy challenging [14, 34]. In addition, they are usually resistant to pharmacological treatment with somatostatin analogs and dopamine agonists. Chemotherapy has been reserved as salvage therapy in aggressive cases although the results have often been disappointing [34]. Novel therapeutic approaches are therefore needed for these tumors.

A prerequisite to identify novel therapeutic targets for human tumors is a good understanding of the pathogenetic mechanisms. The molecular mechanisms underlying the development of PAs in general, and of gonadotroph adenomas in particular, have only been incompletely unraveled and this is in part due to the limited availability of suitable animal models and by the fact that those available are not always representative of human adenomas [27].

We have recently identified a multiple endocrine neoplasia syndrome occurring in a Sprague–Dawley-derived rat strain (named MENX) characterized by the occurrence of spontaneous PAs with complete penetrance. MENX is caused by a biallelic loss-of-function mutation in *Cdkn1b,* encoding the cell cycle inhibitor p27, which results in a highly unstable protein [29, 33]. MENX-associated PAs are histologically and ultrastructurally remarkably similar to human gonadotroph adenomas. They also show high mitotic activity and elevated Ki67 labeling index [26]. Primary cells derived from these tumors are a suitable model for pharmacological studies of PAs [24].

To elucidate the mechanisms associated with the development of human gonadotroph adenomas we exploited the MENX model and performed whole genome transcriptome analysis of the rat tumors and of normal pituitary tissues of wild-type animals. With this approach, we have identified genes differentially expressed in rat PAs that had been previously found dysregulated in human adenomas by array analysis, but not yet further validated. We here show that two such genes (i.e. *CYP11A1* and *NUSAP1*) are highly expressed at both mRNA and protein level in human gonadotropinomas and may represent novel biomarkers of these tumors. In vitro functional assays demonstrated for the first time that the high expression of *Cyp11a1* promotes proliferation/survival of PA cells (gonadotroph- and somatotroph-derived), thereby supporting a role for this gene in pituitary tumorigenesis. We also determined that *Cyp11a1* expression is regulated by the steroidogenic transcription factor SF-1 in gonadotroph cells. Noteworthy, in addition to *NUSAP1*, other genes involved in mitosis were found overexpressed in both rat and human PAs by meta-analysis. If further validated, these genes may represent novel potential targets for the therapy of gonadotropinomas.

## Materials and methods

### Human pituitary tissue samples

Pituitary adenoma samples were obtained from patients at Imperial College and the University of Tübingen at the time of transsphenoidal surgery. All patients consented for research and appropriate ethical approval for the study was obtained. Fragments of the specimens that were not used for diagnostic assessment were snap-frozen at −80 °C. Gonadotroph tumors were defined as demonstrating positive immunostaining for LHβ, FSHβ or αGSU in >5–10 % of cells. Extension to the cavernous sinus and invasiveness of the sellar floor were identified at preoperative magnetic resonance imaging (MRI). The modified Hardy criteria were applied [18]. Post-mortem samples of normal adenohypophysis used for immunohistochemistry (IHC) were obtained from Imperial College. Autopsies were performed within 2–18 h from death and were formalin-fixed and routinely processed to paraffin embedding. Consent for research use of this tissue was obtained from families. RNA of normal pituitary tissues was purchased from BioChain Inc (Hayward, CA, USA).

### Rat pituitary tissue samples

MENX-affected rats develop multifocal pituitary adenomas in the anterior lobe of the gland. Based on hormone expression by immunohistochemistry we could establish that these tumors mainly derive from gonadotroph cells [26]. The lesions become histologically detectable at 4 months of age and progress to become large, mature tumors that efface the gland at 7–8 months. The number of cells expressing LHβ or FSHβ decreases with tumor progression and is negligible in the largest adenomas, while tumors remain positive for the common gonadotropin alpha subunit (αGSU). Rat adenomas show mitotic activity and high Ki67 labeling (average 8 % at 7–8 months), thus resembling aggressive pituitary adenomas. Approximately 10–17 % of the pituitary lesions contain cells expressing growth hormone (GH) or prolactin (PRL) [26]. The pituitary glands used for RNA extraction (upon microdissection) derived from rats aged 7–8 months having large tumors approximately 1–2 mm in size.

### RNA isolation and microarray preparation

Rat RNA was extracted from macrodissected pituitary tissues and processed for array analysis as reported [30]. Amplified cDNA (2 μg) was hybridized on Affymetrix Rat Gene 1.0 ST arrays (Santa Clara, CA, USA) containing about 26 k gene-level probe sets. Staining and scanning was done according to the Affymetrix expression protocol. RNA was extracted from frozen human pituitary tumor samples following standard protocols [30].

### Biostatistical and bioinformatic analysis

Expression console (Affymetrix) was used for quality control and to obtain annotated normalized RMA gene-level data (standard settings including median polish and sketch-quantile normalization). Statistical analyses and heat maps were generated in CARMAweb [36]. Genewise testing for differential expression was done employing *t* test and Benjamini-Hochberg multiple testing correction (FDR < 5 %). Genes with fold-changes >2× were further analyzed using GePS software (Genomatix Software GmbH, Munich, Germany). Signaling pathways containing, or regulated by, differentially expressed genes were identified using the Ingenuity Pathway Analysis (IPA) library of canonical pathways. Array data were submitted to Gene Expression Omnibus (GSE23207). The comparison of rat and human datasets was done in Venny (http://bioinfogp.cnb.csic.es/tools/venny/index.html) based on the match of human genes to rat probe set IDs via rat Entrez IDs provided by Affymetrix. Human Entrez IDs of regulated genes were matched to rat Entrez IDs mainly based on the NCBI homologene database, supplemented with information provided by Ingenuity pathway software. Human datasets were taken from publications: Morris et al. [32], 1,723 probe sets with fold-changes >1.5×; Moreno et al. [31], Supplementary Table 2,297 probe sets with fold-changes >2×) or calculated from.cel files; Michaelis et al. [28], Acc. Number GSE26966, analysis as described above, 3,558 probe sets with ratios >2×. All human datasets were done with Affymetrix arrays and human Entrez IDs of regulated probe sets were taken from annotations provided by Affymetrix. For pairwise comparisons of rat and human datasets, probe set based gene lists from the Venn diagram were further processed: for redundant entries only the entry with the highest ratio in rat was kept, and datasets were filtered for the same direction of regulation in rat and human datasets.

### Cell culture, treatments and assays

GH3 and Y1 cells were purchased from the ATCC and grown in DMEM supplemented with 10 % (v/v) FBS and 1 % (v/v) penicillin–streptomycin. The murine gonadotroph cell line LβT2 was kindly provided by P. Mellon (University of California, San Diego, USA) and was grown in DMEM + GlutaMAX™-I plus 4.5 g/l d-glucose and pyruvate with 10 % (v/v) FBS and 1 % (v/v) penicillin–streptomycin. Primary pituitary tumor cells were isolated from mutant rat pituitaries and grown as previously described [24]. Media, serum and supplements were from Gibco/Invitrogen (Karlsruhe, Germany). Transfection with siRNA oligos was performed as previously reported [24]. For infections, GH3 and primary tumor cells were plated on 96-well plates and 24 h later cells were infected by lentiviral vectors expressing the green fluorescence protein (GFP) or GFP and shRNA against *Cyp11a1*. Virus production and lentiviral infection were performed according to previous protocols [2]. WST-1 colorimetric assay (Roche, Mannheim, Germany) for cell viability was performed 72 h after infection according to the manufacturer’s recommendations.

Apoptosis was measured by assessing the activity of caspase-3/7 using Caspase-Glo^®^ 3/7 Assay kit (Promega, Mannheim, Germany). Primary cells or transfected GH3 cells were plated in 96-well plates and 36 h later were infected with sh-*Cyp11a1* or mock GFP lentiviral vectors. Forty-eight h after treatment, caspase-3/7 enzymatic activity was assessed with a proluminescent caspase-3/7 substrate which contains the tetrapeptide sequence DEVD. Luminescence was measured using a luminometer (Berthold, Bad Wildbad, Germany).

For treatment with ethyl 2-[[2-[2-[(2,3-dihydro-1,4-benzodioxin-6-yl)amino]-2-oxoethyl]-1,2-dihydro-1-oxo-5-isoquinolinyl] (IsoQ) (Tocris Bioscience, Bristol, UK), 2.5 × 10^4^ primary rat pituitary cells (2.5 × 10^4^ cells/well) or LβT2 cells (1 × 10^4^ cells/well) were seeded into 96-well plates and allowed to settle for 36 h. IsoQ was dissolved in DMSO and serial dilutions (10 μM–10 nM) were prepared in culture medium. Treated cells were incubated with IsoQ for 24 and 48 h, while control cells with 0.01 % (v/v) DMSO. Cell viability was assessed using the WST-1 assay as above.

### Quantitative TaqMan RT-PCR

Quantititative RT-PCR was performed using TaqMan inventoried primers and probes for the genes reported in the article and for the rat beta 2-microglobulin gene, mouse beta 2-microglobulin gene or human TBP gene as internal controls (Applied Biosystem, CA, USA). RNA was extracted and TaqMan assays were set up as previously reported [30].

### RNA interference

The specific *Cyp11a1* shRNA sequence was cloned into the pSUPER lentiviral vector. The sequence of the complementary oligonucleotides is: sh-*Cyp11a1* Fw GATCCGGATGTTGGAGGAGATCGTTTCAAGAGAACGATCTCCTCCA ACATCCTTTTTG; Rev: AATTCAAAAAGGATGTTGGAGGAGATCGTTCTCTT GAAACGATCTCCTCCAACATCCG.

### Immunohistochemistry and Immunofluorescence

Immunohistochemistry (IHC) staining was performed on an automated immunostainer (Ventana Medical Systems, Tucson, AZ, USA) according to the manufacturer’s protocols with minor modifications, as previously described [30]. Primary antibodies were raised against Ass1 (Sigma, Louis, MO, USA); Ki-67 (Clone B56, Dako, Hamburg, Germany); rat P450scc (Abcam, Abcam, Cambridge, UK); human P450scc (Santacruz, Heidelberg, Germany); NuSAP (Proteintech, Chicago, IL, USA). Antibodies were diluted in Dako REALTM antibody diluent (Dako). The supersensitive detection system (BioGenex, Munich, Germany) was used and the immunoreaction was developed in the diamino-benzidine (DAB) supplied with the kit (Vector lab, Burlingame, CA). Positive controls were included in each batch. To score the P450scc staining, both the intensity of the staining and the number of positive tumor cells were taken into consideration. For number of positive cells, we used a score: 0 = 0 % (or <1 %) positive cells; 1 = up to 25 % positive cells; 2 = up to 50 %; 3 = up to 75 %; 4 = up to 100 %. For staining intensity, a 4-scaled scoring system was used: 0 = negative, 1 = weak, 2 = moderate, 3 = strong. These values were then multiplied to obtain ‘immunoreactivity scores’ (IRS). Ki67 and NuSAP nuclear immunoreactivity was determined semiquantitatively and was indicated as the percent of positive cells against all neoplastic cells in the section examined (Labeling Index, LI). Images were recorded using a Hitachi camera HW/C20 installed in a Zeiss Axioplan microscope with Intellicam software (Carl Zeiss MicroImaging GmbH, Gottingen, Germany).

For immunofluorescence (IF), we used the primary antibodies used for IHC and secondary anti-mouse Alexa Fluor^®^ 555 Conjugate (Cell Signaling) or anti-rabbit FITC-conjugated (Invitrogen, Darmstadt, Germany) antibodies as reported [26]. Sections were then analyzed with a Zeiss Axiovert 200 epifluorescence microscope including Apotome unit (Carl Zeiss MicroImaging GmbH).

### Statistical analysis

Results of the cell viability assays are shown as the mean of values obtained in independent experiments ± SEM (standard error of the mean). A paired two-tailed Student’s *t* test was used to detect significance between two series of data and *P* value (*P*) < 0.05 was considered statistically significant.

## Results

### Genetic signature of MENX-associated pituitary adenomas

In order to unravel the genetic changes associated with pituitary adenoma (PA) formation, we performed whole genome transcriptome analysis of 16 individual pituitary lesions from 11 adult MENX mutant rats (8 months of age) and compared them with 5 normal pituitaries. Using a >twofold-change cut-off for gene expression changes, 487 probe sets appeared up-regulated in adenomas versus normal wild-type gland, and 400 were down-regulated (Supplementary Fig. 1 and Dataset 1). The relative high number of differentially expressed transcripts is not unexpected since normal pituitary is composed of multiple cell types of which only 10–20 % are gonadotroph cells. Gene ontology (GO) term enrichment analysis showed that genes related to cell cycle (*P* = 9.85E−09), development (*P* = 1.2E−06), cell differentiation (*P* = 1.36E−06), cell proliferation (*P* = 2.53E−07), and lipid metabolism (*P* = 9.43E−04) are overrepresented in our dataset (ratios >twofold, Table 1). Genes encoding proteins involved in cell cycle regulation and especially in mitosis such as *Aurka*, *Bub1*, *Bub1b*, *Ccna1*, *Ccnb1*, *Ccnb2*, *Ccne1*, *Cdc2*, *Cdc20*, *Cdkn3*, *Kif4*, *Kif11*, *Nusap1*, *Prc1*, as well as genes involved in pituitary development or adenohypophyseal cell differentiation, such as *Dax*-*1/Nr0b1, Egr1*, *Fgfr2, Neurod1, Notch2, Nr5a1, Pou1f1, Tbx19* [6, 10, 23] were differentially expressed in rat PAs (Table 1). Array data mining found that genes such as *Ccnb1*, *Igfbp3*, *Nusap1, Pttg1*, *Racgap1*, *Top2a*, *Tpx2,* which are associated with the aggressive behaviour of various human tumors, including PAs, and proto-oncogenes such as *Ect2*, *Kit*, *Kras*, *Lyn*, *Ret* are up-regulated in rat adenomas [12, 15, 42, 43]. This result is in agreement with the elevated proliferative activity of the tumors [26]. Interestingly, also genes belonging to the category “lipid metabolic process” were significantly overrepresented in the rat adenomas, and several of them are known targets of the transcription factor steroidogenic factor 1 (SF-1) (see below).

**Table 1:** Enriched GO categories by GePS software from Genomatix

GO-term	GO-term ID	*P* value	Observed/expected	Number of genes in GO	Selected dysregulated genes
*Enriched terms within the up-regulated genes*					
Cell cycle	GO: 0007049	9.85E−09	42/16	667	Aurka, Bmp7, Bub1, Bublb, Ccnbl, Ccnb2, Cdc2, Cdc20, Cdknla, Cdkn2c, Cdkn3, Cenpf, Cited2, Id2, Nusapl, Pttgl
Developmental process	GO: 0032502	1.20E−06	98/62	2,597	Angpt2, Ass1, Bmp7, Cdkn2c, Cited2, Cyp11a1, Fgfr2, Id2, Igfbp3, Igfbp7, Lyn, Neurod1, Nos1, Nr0b2, Nr0b1, Nr5a1, Ret, Sox11
Cell differentiation	GO: 0030154	1.36E−06	63/34	1,431	Angpt2, Apoe, Ar, Bag1, Bmp7, Ccnb1, Cdc2, Cdkn2c, Cenpf, Cited2, Cyp11a1, Fgfr2, Id2, Igfbp3, Kit, Kras, Lyn, Neurod1, Neurod4, Nr0b1, Nr5a1, Racgap1, Ret, Sox11, Top2a, Tshr, Vegfa
Cell proliferation	GO: 0008283	2.53E−07	45/20	832	Apoe, Ar, Bub1, Ccna2, Ccnb1, Cdc2, Cdc20, Cdkn1a, Cdkn1c, Cdkn2c, Cenpf, Fgfr2, Gnrhr, Id2, Igfbp3, Kit, Kras, Lyn, Pttg1, Racgap1, Scarb1, Sox11, Tac1, Vegfa
Lipid metabolic process	GO: 0006629	9.43E−04	33/19	772	Apoe, Apof, Cyp1b1, Cyp11a1, Cyp11b1, Cyp11b2, Fdxr, Nr0b1, Scarbl
*Enriched terms within the down-regulated genes*					
Developmental process	GO: 0032502	7.73E−10	69/34	2,597	Egr1, Notch2, Nr4a3, Sema3e, Dlk1, Angptl, Nr4a2, Ghrhr, Adcyap1r1, Pou1f1, Tbx19
Negative regulation of cell proliferation	GO: 0008285	9.29E−03	9/4	271	Nfib, Notch2, Gjb6, Wfdd, Rbp4, Gpc3, Cdk6, Msx1, Slit2

Genes encoding pituitary hormones were down-regulated in rat adenomas, with the exception of *Cga* encoding the common alpha subunit of the gonadotropins (αGSU), which was highly expressed in all adenomas (Supplementary Fig. 1). These results are in keeping with previous immunohistochemical stainings showing that the large, older rat pituitary lesions do not express any hormone subunit except αGSU [26].

Transcription factor analysis using Ingenuity Pathway Analysis (IPA) predicted the activation of several upstream transcription factors, including *Nr5a1* (*z*-score = +2.34; *P* = 5E−08) encoding SF-1 (Supplementary Table 1). The genes encoding the gonadotropin hormone subunits LHβ and FSHβ, which are transcriptionally regulated by SF-1 in gonadotroph cells, are down-regulated in the large rat tumors, as mentioned above. In contrast, several downstream targets of SF-1 were found highly expressed in the adenomas. While the mRNA level *Nr5a1* was up-regulated 2.7-fold in the adenomas (Dataset 1), its downstream targets were overexpressed up to 23-fold in the tumors, including *Angpt2*, *Cyp11a1*, *Cyp11b1, Cyp11b2*, *Giot1*, *Gnrhr*, *Nos1*, *Nr0b1*/*Dax1*, *Nr0b2*, *Scarb1*. Also *Fgf13* and *Col18a1* were up-regulated, as reported in adrenal cancer cells [8], and so was *Cited2*, encoding for a co-activator of SF-1 during adrenal development [40]. Altogether, these data suggest that the activity of SF-1 is enhanced in rat PAs (Supplementary Fig. 2a). Further support to the activation of SF-1 in the rat tumors came from the validation of *Cyp11b1* and *Cyp11b2* overexpression in independent adenoma samples (Supplementary Fig. 2b). Several of the SF-1 targets mentioned above play a physiological role in the synthesis or transport of steroid hormones in steroidogenic tissues and have not so far been implicated in adenohypophyseal tumorigenesis.

**Fig. 1:**
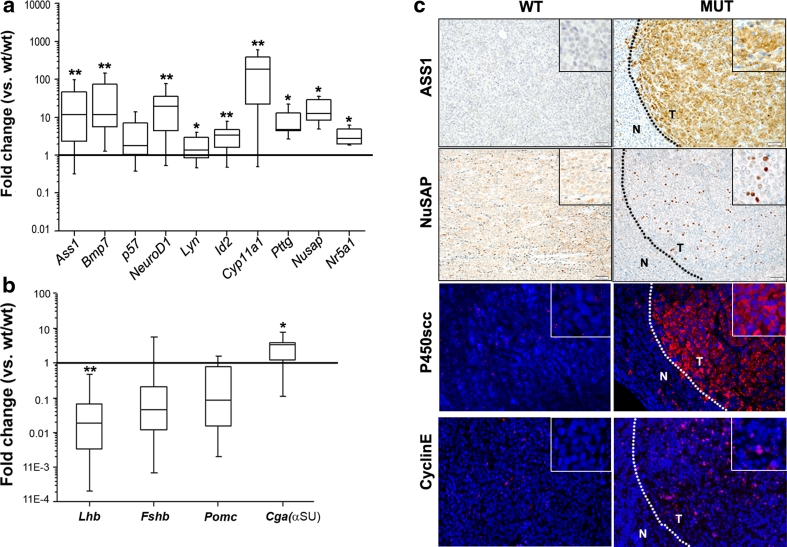
Validation of selected genes by qRT-PCR and by IHC on rat PA tissues. **a**, **b** RNA was extracted from 11 macrodissected pituitary tumors of adult mutant rats and from five normal pituitaries of wild-type (wt/wt) rats. qRT-PCR was performed using TaqMan primer and probe sets specific to **a** rat genes up-regulated by array analysis: *Ass1*, *Bmp7*, *p57*, *NeuroD1*, *Lyn*, *Id2*, *Cpy11a1*, *Pttg*, *Nusap,* *Nr5a1* and **b** rat genes down-regulated by array analysis: *Lhβ*, *Fshβ*, *Pomc*, *Cga* (αGSU). The relative mRNA expression level of the target genes was normalized for input RNA using the housekeeping β2-microglobulin gene and a calibrator RNA always run in parallel and was calculated with the 2^−ΔΔCt^ formula. The obtained relative value was normalized against the average level in normal pituitary, arbitrarily set to 1. The boundary of the *box* closest to zero indicates the 25th percentile, the *line* within the *box marks* the median, and the boundary of the *box* farthest from zero indicates the 75th percentile. *Error bars* *above* and *below* the *box* indicate the 99th and 1th percentiles. *P < 0.05, **P < 0.001. **c** Formalin-fixed, paraffin-embedded (FFPE) pituitaries from adult wild-type (WT) (*left*) and mutant (MUT) (*right*) rats were used. IHC was performed using antibodies against ASS1 and NuSAP and counterstained with hematoxylin. Immunofluorescence was performed with antibodies against P450scc or Cyclin E and nuclei were counterstained with DAPI. *T* tumor area, *N* normal adjacent area. Original magnification: ×200; *insets*: ×400

Real-time quantitative RT-PCR (qRT-PCR) for 9 selected overexpressed genes was performed on 11 additional individual pituitary adenomas to validate the array data (Fig. 1a). These tumors were compared with five pituitaries from wild-type rats. *Nr5a1* (Sf-1) and *Cga* (αGSU) were included as controls because the encoded proteins are expressed in these tumors [26], while the other transcripts were chosen because they have interesting functions and they span a broad range of increased fold-changes, from ~3-fold to >20-fold. The results confirmed the up-regulation of these genes in rat PAs, with *Cdkn1c* (p57) showing a trend toward higher levels in the tumors versus normal pituitary which did not reach the statistical significance (Fig. 1a). qRT-PCR also confirmed the down-regulation of *Pomc*, *Lhb*, *Fshb* in rat adenomas (Fig. 1b).

The differential expression of selected genes was further validated with immunohistochemistry (IHC) and immunofluorescence (IF). Stainings for argininosuccinate synthetase 1 (Ass1), NuSAP, Cyclin E, P450scc (encoded by *Cyp11a1*) showed higher Ass1 and P450scc levels in tumors compared with non-tumorous pituitary tissue of mutant rats or wild-type rat pituitaries (Fig. 1c). Similarly, NuSAP- and CyclinE-positive cells were found in higher number in adenomas compared with surrounding non-tumorous gland, or with normal pituitaries (Fig. 1c).

### Dysregulated pathways and genes previously associated with human pituitary tumors

Some of the pathways identified as dysregulated in rat PAs by the IPA software have also been implicated in human PAs by proteomics profiling, and include cell cycle, lipid metabolism and endocrine system development [45]. In addition, the homologues of several genes up-regulated in the rat tumors had also been already implicated in human non-functioning/gonadotroph PAs. Examples are *ECT2*, *NEUROD1*, *PTTG1*, *VEGFA* and the oncogene c-*fos* [22, 25, 31, 32]. *NOS1* and *DAX1,* regulated by and/or regulating the transcription factor SF-1, are highly expressed in human gonadotroph adenomas [20, 39]. Androgen receptor (AR) is expressed in 74 % of gonadotropinomas [37], and *DLK1* is silenced in non-functioning adenomas, while it is highly expressed in functional adenomas [1].

**Fig. 2:**
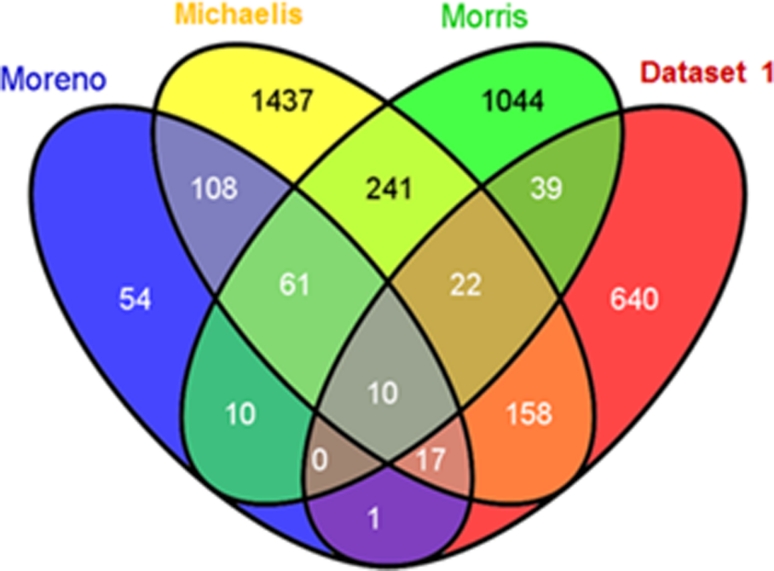
Overlap of differentially expressed genes. Venn diagram displaying the overlap among lists of differentially expressed genes from gene expression studies in human gonadotroph/non-functioning PAs [28, 31, 32] and rat PAs (Dataset 1). For details, see “Materials and methods”

Studies comparing the transcriptome of human gonadotroph adenomas with that of normal pituitary tissues [1, 9, 11, 28, 31] or of non-invasive versus invasive gonadotroph adenomas [12, 19] have been performed. We therefore compared our Dataset 1 with the available lists of significantly differentially expressed genes in human non-functioning/gonadotroph adenomas. Examples of concordantly dysregulated genes in both species are reported in Supplementary Table 2. Remarkably, the degree of overlap of genes differentially expressed among different human sample sets is similar to the overlap between rat and human datasets, as estimated using Venn diagrams (Fig. 2). The 10 genes differentially expressed in all the datasets we compared are: *Ptprk, Atp8a1, Lgals3, Nr4a2, Pon3, Fos, Angpt1, Amigo2, Rcan2, Maob* (for details, please refer to “Materials and methods”). We found several common dysregulated genes in the rat tumors and in the dataset recently published by Michaelis et al. [28] analyzing 14 human gonadotroph adenomas. Among the genes dysregulated in both rat and human adenomas, we found genes associated with pituitary function or pituitary tumorigenesis and mentioned earlier in the article, such as *AR*, *CCNB1*, *DLK1*, *ECT2*, *FOS*, *NEUROD1*, *NR0B1/DAX1*, *VEGF*. Genes encoding transcription factors and hormones important in other pituitary cell lineages (i.e. *POU1F1/PIT*-*1, TBX19, GH, PRL, POMC*) are down-regulated in rat and human gonadotroph-derived adenomas, as expected. *BAG1*, coding for an anti-apoptotic protein, was found overexpressed in human non-functioning PAs by array analysis [32], and is also up-regulated in MENX-associated adenomas. Similarly to the rat tumors, several downstream targets of SF-1 were found to be highly expressed in human gonadotroph adenomas by array analysis (Supplementary Fig. 3). Interestingly, several genes dysregulated in both human and rat array datasets, although not yet evaluated in PAs, have been shown to play a role in other human malignancies, such as *BUB1*, *BUB1B, GALNT12, GPC3, HMMR, KIF4, NR4A3, NUSAP1, PDE4B, PRC1* and thus warrant follow-up studies.

### CYP11A1 and NUSAP1 are overexpressed in human PAs

**Fig. 3:**
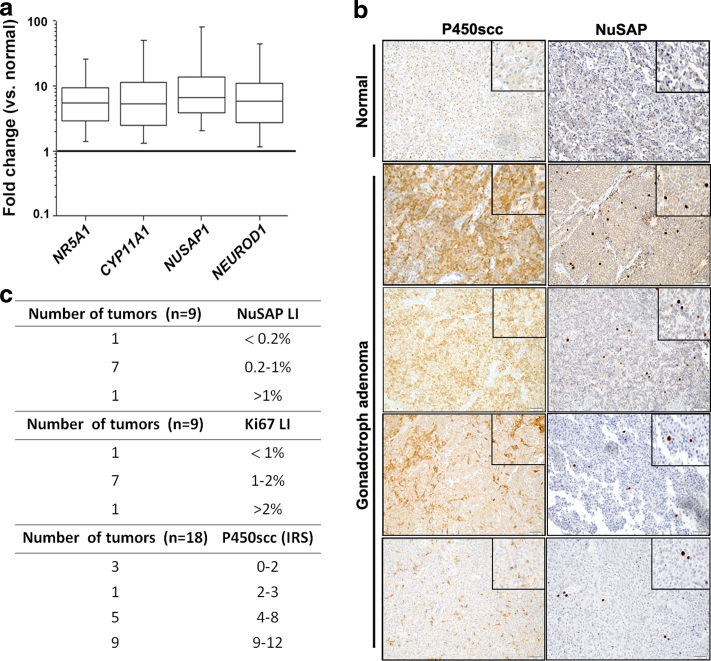
Overexpression of *CYP11A1* and *NUSAP1* and their encoded proteins in human gonadotroph tumors. **a** RNA was extracted from frozen pituitary tumors obtained after transsphenoidal surgery. qRT-PCR was performed using TaqMan primer and probe sets specific to human *NR5A1*, *CYP11A1*, *NUSAP1* and *NEUROD1*. The relative mRNA expression level of the target genes was normalized for input RNA using human *TBP* gene expression (housekeeping gene) and a calibrator human brain RNA always run in parallel and was calculated with the 2^−ΔΔCt^ formula. The obtained relative value was normalized against the average expression of normal pituitary arbitrarily set to 1. The boundary of the *box* closest to zero indicates the 25th percentile, the *line* within the *box marks* the median, and the boundary of the *box* farthest from zero indicates the 75th percentile. *Error bars* *above* and *below* the *box* indicate the 99th and 1th percentiles. **b** Human normal pituitary (*top*) and gonadotroph adenoma samples were used. IHC was performed using antibodies against P450scc (*left*) or against NuSAP (*right*) and counterstained with hematoxylin. Original magnification: ×200; insets: ×400. **c** Scoring of the expression of NuSAP, Ki-67 and P450scc in a series of human gonadotroph adenomas

Among the genes overexpressed in the rat pituitary adenomas, predicted to be up-regulated in at least 2 of the 3 human expression array studies by data mining (Supplementary Table 2, in bold), and never before associated with pituitary adenomas, are *Cyp11a1* and *Nusap1*. Thus, these two genes were selected for further analyses. *Cyp11a1* encodes P450scc, the mitochondrial enzyme that catalyzes the conversion of cholesterol to pregnenolone in steroidogenic tissues. *Nusap1* encodes a protein that plays a role in spindle microtubule organization and is necessary for cell division [35]. To verify whether these genes are truly up-regulated in human gonadotroph adenomas, we performed qRT-PCR in a series of 39 samples. *NR5A1* and *NEUROD1* were also included in the analysis as they are expected to be up-regulated in these tumors. We found that *CYP11A1* was up-regulated in 77 % (27 out of 35) and *NUSAP1* in 95 % (37 out of 39) of the human PAs (Fig. 3a). *NR5A1* and *NEUROD1* were overexpressed in 82 % of the cases.

To determine whether gene up-regulation translates into higher levels of the encoded protein, we obtained formalin-fixed, paraffin-embedded (FFPE) tissue blocks from 18 of the adenomas analyzed by qRT-PCR, and stained them for P450scc and NuSAP. Normal anterior pituitary cells had only weak cytoplasmic positivity for P450scc in sparse hypophyseal cells, while P450scc staining was more intense, although often uneven, in adenomas (Fig. 3b). Immunoreactivity score (IRS) were calculated and showed that 13 of 18 cases (72 %) had a IRS ≥6, indicating moderate to strong staining in up to 75 % of the tumor cells, and 5 cases (28 %) showed weaker staining (IRS ≤4) (Fig. 3c). Of the human gonadotropinomas used for IHC, 9 were invasive and 8 non-invasive. No significant difference in the degree of positivity for P450scc was observed between the two groups of tumors.

**Fig. 4:**
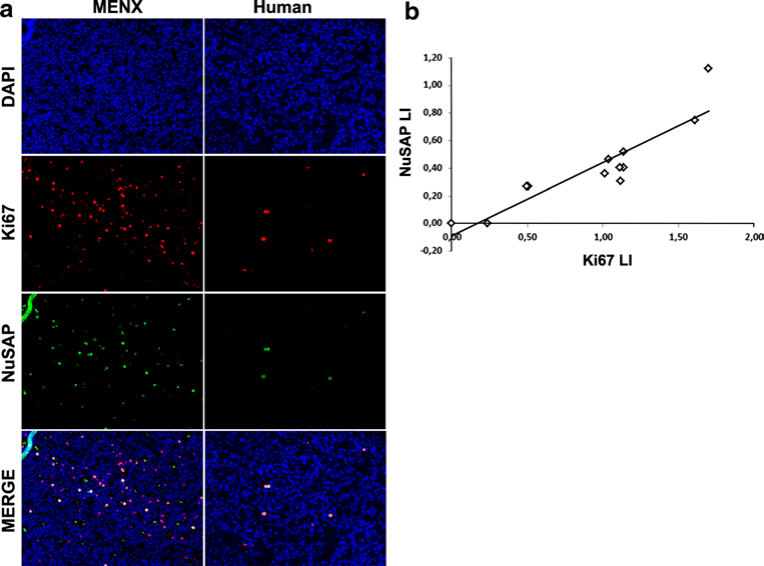
Expression of Ki-67 and NuSAP in rat and human pituitary adenoma cells. **a** Example of immunofluorescent staining with antibodies against Ki-67 (*red*) and NuSAP (*green*) performed on FFPE pituitary tumor tissues from a MENX rat (MENX) and from a patient with gonadotroph adenoma (Human). Nuclei were counterstained with DAPI. Only the tumor area is shown. Original magnification: ×100. **b** Correlation between NuSAP and Ki-67 labeling index (LI) in 12 human gonadotroph adenoma samples

We also performed IHC to detect NuSAP expression and we could detect tumor cells having a strong, nuclear staining in every human PA sample (Fig. 3b). Being NuSAP associated with mitosis, we determined the relationship between its expression and that of the Ki67 antigen, an established marker of cell division. Quantification of the IHC results demonstrated that Ki-67-positive cells were more numerous than NuSAP-positive cells (Fig. 3c). NuSAP-positive pituitary tumor cells always co-expressed Ki-67 (Fig. 4a). Co-expression of the two markers was also seen in rat PAs, which have elevated proliferation rates (Fig. 4a) [26]. A good correlation between the Labeling Index (LI) of NuSAP and of Ki-67 was seen in the human tumor tissue samples (*R* ^2^ = 0.8131) (Fig. 4b).

### Cyp11a1 plays a pro-proliferative role in tumorigenesis

We have established that *Cyp11a1* is highly expressed in rat and human gonadotroph tumors at mRNA and protein levels. To clarify the role of this gene in tumorigenesis, we first used Y1 cancer cells derived from a steroidogenic tissue and expressing *Cyp11a1* at high level. siRNA-mediated knock-down of *Cyp11a1* reduced the ability of Y1 cells to proliferate when compared with cells transfected with scrambled siRNA oligos (Supplementary Fig. 4b). Efficient knock-down of the *Cyp11a1* gene was verified by qRT-PCR (Supplementary Fig. 4a). Encouraged by these findings we pursued the role of *Cyp11a1* overexpression in pituitary tumorigenesis. We used somatotroph adenoma-derived GH3 cells, which express relatively high levels of *Cyp11a1* (Supplementary Fig. 5), to evaluate the effect of modulating gene expression on pituitary tumor cell proliferation. We found that siRNA-mediated silencing of *Cyp11a1* reduced GH3 cells proliferation, while scrambled siRNA oligos elicited no effect (Supplementary Fig. 4c, d).

**Fig. 5:**
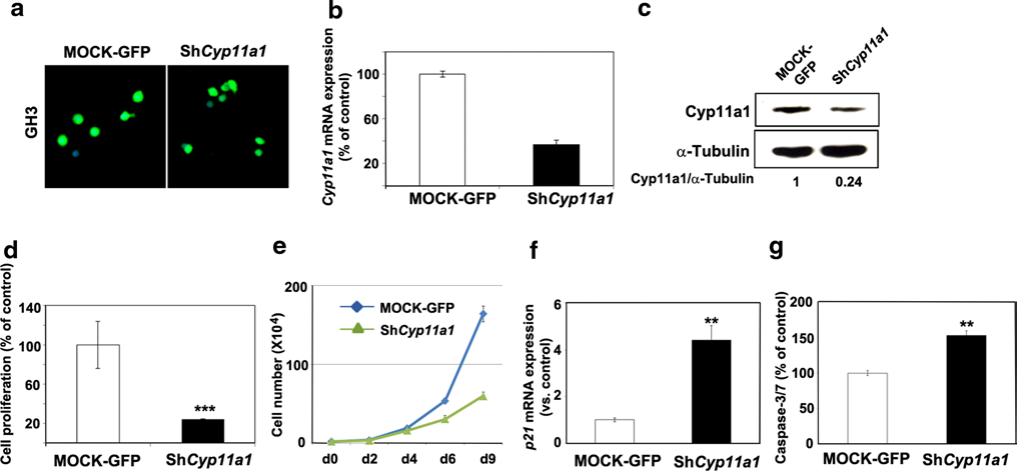
Effect of *Cyp11a1* on tumor cell proliferation. **a** Infection of GH3 cells with lentiviral vectors expressing shCyp11a1-GFP or GFP only (MOCK). **b** GH3 cells were infected with the lentiviral vectors as in “**a**” and the level of *Cyp11a1* was analyzed by qRT-PCR 72 h later. **c** In samples parallel to “**b**”, proteins were extracted, and Western blotting was performed to monitor Cyp11a1 expression. The ratios of the band intensities for P450scc versus α-tubulin, normalized against the ratio in the MOCK control (ratio = 1), is indicated. **d** In samples parallel to “**b**”, cell proliferation was assessed 72 h after infection using the WST-1 assay. Data were analyzed independently with six replicates each and were expressed as the mean ± SEM. **e** Growth curve of GH3 cells infected with lentiviral vectors expressing shCyp11a1-GFP or GFP only (MOCK). Cells were trypsinized, stained with Trypan Blue and viable cells were counted. Values are the mean of 3 independent experiments ± SD. **f** In samples parallel to “**d**”, the expression of p21 was assessed by qRT-PCR as indicated in the legend of Fig. 1, and is reported relative to the expression level in mock-transfected cells arbitrarily set to 1. **g** In samples parallel to “**d**”, caspase 3/7 activity was measured to monitor apoptosis 72 h post-infection. Data were analyzed independently with six replicates each and were expressed as the mean ± SEM. **P < 0.01, ***P < 0.001, versus MOCK

qRT-PCR showed that rat primary PA cells maintain the high *Cyp11a1* expression level observed in the tumor tissues from which they derive (Supplementary Fig. 5), and thus represent a suitable model for analyzing the effect of gene knock-down. As these cells were difficult to transfect, we generated a lentiviral vector expressing both a small hairpin (sh) RNA molecule directed against rat *Cyp11a1* and the green fluorescent protein (GFP) to monitor infection efficiency (ca. 80 %, data not shown). Two RNA sequences were tested but only one gave a consistent and robust gene down-regulation and was then used on further experiments (data not shown). The sh-*Cyp11a1* lentiviral vector was first tested in GH3 cells (Fig. 5a), where it caused a very strong decrease in *Cyp11a1* expression (Fig. 5b, c) and, concomitantly, a reduction in cell proliferation when compared with mock infected cells (GFP) (Fig. 5d). In growth curve experiments, viral-mediated *Cyp11a1* knock-down decreased the growth rate of GH3 cells, while infection with the mock vector had not effect on cell growth (Fig. 5e).

**Fig. 6:**
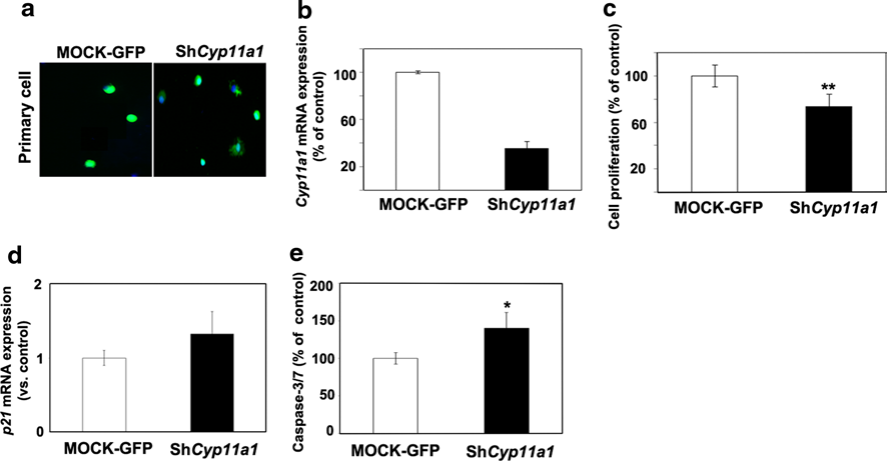
Effect of *Cyp11a1* on primary pituitary tumor cell proliferation. **a** Infection of rat primary pituitary tumor cells with lentiviral vectors expressing shCyp11a1-GFP or GFP only (MOCK). **b** Primary pituitary tumor cells from three mutant rats were infected with the lentiviral vectors as in “**a**” and the level of *Cyp11a1* was analyzed by qRT-PCR as indicated in the legend of Fig. 1, and is reported relative to the expression level in mock-transfected cells arbitrarily set to 100. **c** In samples parallel to “**b**”, cell proliferation was assessed 72 h after infection using the WST-1 assay. Data were analyzed independently with six replicates each and were expressed as the mean ± SEM. **d** In samples parallel to “**c**”, the expression of p21 was assessed by qRT-PCR as indicated in the legend of Fig. 1, and is reported relative to the expression level in mock-transfected cells arbitrarily set to 1. **e** Caspase 3/7 activity was measured in samples in parallel to “**c**” to monitor apoptosis in GH3 cells 72 h post-infection. *P < 0.05, **P < 0.01 versus MOCK

To determine what caused the reduction in the proliferation capacity of GH3 cells, we checked for the induction of cell cycle inhibitors following *Cyp11a1* gene knock-down. We determined the level of expression of p16, p18 and p21 after infection by qRT-PCR and we found that while the amount of p16 and p18 transcripts does not change (data not shown), p21 expression increases significantly upon reduction of *Cyp11a1* levels (Fig. 5f). We also assessed caspase-3/7 activity as a measure of apoptosis in GH3 cells infected with GFP or with sh-*Cyp11a1*. Caspase-3/7 activity is higher in cells infected by sh-*Cyp11a1* compared with mock infected cells, suggesting that this process might also explain part of the reduced cell growth (Fig. 5g). We then went back to our original model (MENX rats) and established primary PA cultures from three independent mutant rats. Cells were infected with sh-*Cyp11a1* vector, or with the mock GFP vector (Fig. 6a) and cell viability was measured. Upon infection of the primary cells with sh-*Cyp11a1* vector, but not with mock vector, we observed a reduction in *Cyp11a1* expression (Fig. 6b), which was accompanied by a decrease in cell viability (−30 % versus control GFP) (Fig. 6c). Similarly to what we observed in GH3 cells, also in primary rat PA cells there was an increase in both p21 expression and caspase-3/7 activity, although less pronounced than in GH3 cells (Fig. 6d, e). In conclusion, high expression of *Cyp11a1* associates with increased proliferation and survival not only in adrenocortical carcinoma cells (Y1), but also in rat pituitary tumor cells from different lineages (somatotroph and gonadotroph).

**Fig. 7:**
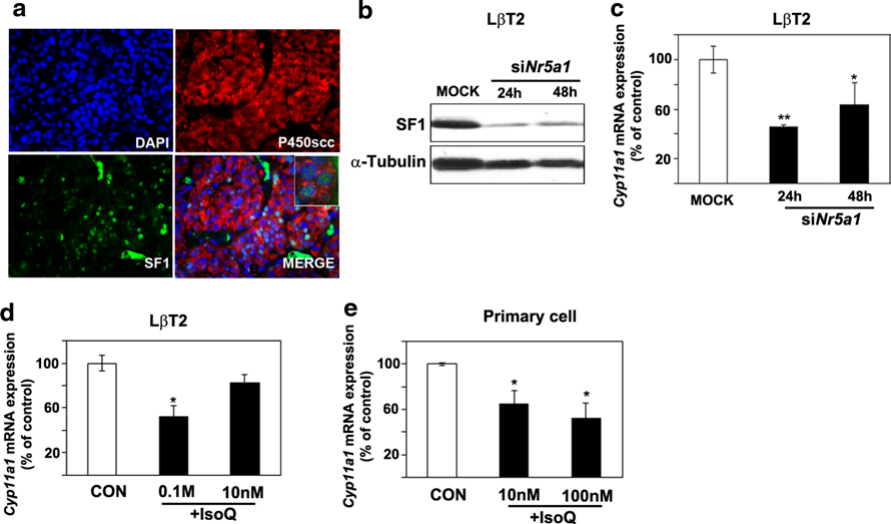
Co-localization of P450scc and SF-1 in rat PAs and reduction of *Cyp11a1* expression following down-regulation or inhibition of SF-1 in gonadotroph cells. **a** Immunofluorescent staining with antibodies against P450scc (*red*) and SF-1 (*green*) was performed on FFPE pituitary tumor tissues from MENX mutant rats. Nuclei were counterstained with DAPI. The *inset* in MERGE shows the co-localization of SF-1 and P450scc in rat tumor cells. Original magnification: ×200; inset: ×400. **b** LβT2 cells were transfected with scrambled (MOCK) or siRNA oligos against the mouse *Nr5a1* gene (siNr5a1). SF-1 and α-tubulin expression levels were assessed by Western blotting 24 and 48 h after transfection. **c** In samples parallel to “**b**”, *Cyp11a1* expression level was assessed by qRT-PCR as indicated in the legend of Fig. 1, and is reported relative to the expression level in mock-transfected cells arbitrarily set to 100. **d** LβT2 cells were treated with different concentrations of the SF-1 inhibitor IsoQ (0.1 M and 10 nM) or *left* untreated (CON). After 24 h, we determined *Cyp11a1* expression levels as in “**c**”. **e** Primary pituitary tumor cells from mutant rats (*n* = 3) were incubated with IsoQ as in “**d**”. After 24 h, we assessed *Cyp11a1* expression levels by qRT-PCR as in “**c**”. Data from primary cultures were analyzed independently with six replicates each and were expressed as the mean ± SEM. *P < 0.05, ***P < 0.001 versus MOCK or CON

Rat PA cells express both *Nr5a1* and *Cyp11a1* and, noteworthy, in these cells SF-1 and P450scc co-localize, as determined by dual co-immunofluorescence (Fig. 7a). SF-1 regulates *Cyp11a1* transcription in steroidogenic cells but whether it also does so in adenohypophyseal cells is not known. Therefore, we silenced *Nr5a1* expression by means of siRNA oligos in gonadotroph LβT2 cells and then assessed the level of *Cyp11a1* mRNA by qRT-PCR. As experimental control, we used again Y1 adrenocortical cancer cells, where *Nr5a1* gene down-regulation caused a reduction of *Cyp11a1* mRNA expression (Supplementary Fig. 6). We observed that knock-down of *Nr5a1* in LβT2 cells, as demonstrated by western blotting (Fig. 7b), associates with a decrease in *Cyp11a1* expression (Fig. 7c). To further confirm that SF-1 transcriptional activity plays a role in regulating *Cyp11a1* expression in these cells, we treated LβT2 cells with a small molecule inhibitor of SF-1, isoquinolinone (IsoQ) [8]. This compound caused a significant dose-dependent reduction of *Cyp11a1* expression (Fig. 7d). Similar results were also obtained exposing rat primary PA cells (with high endogenous levels of *Cyp11a1*) to IsoQ (Fig. 7e). Altogether, these data provide indirect evidence that SF-1 is involved in regulating *Cyp11a1* expression in gonadotroph cells.

## Discussion

We recently demonstrated that PAs developing in MENX-affected rats are pathologically similar to human gonadotroph adenomas [26]. The current study confirms this observation and shows that common genetic signatures exist between MENX-associated and human gonadotropinomas. In addition, novel putative markers of human PAs were identified, suggesting that MENX-associated neoplasms may be exploited as to gene discovery.

In silico analysis of gene expression array data of human gonadotroph adenomas identified several genes differentially expressed between tumors and normal pituitaries in both patients and MENX rats. An example of a gene down-regulated in both rats and human PAs is *Dlk1,* encoding a non-canonical ligand of Notch. *DLK1* was found to be selectively silenced in NFPAs but not in other types of PA [1, 5]. *DLK1* is located in the DLK1/MEG3 imprinted locus on human chromosome 14q32.3 and *MEG3*, coding for a maternally imprinted long noncoding RNA with tumor suppressive function, was also found to be specifically silenced through methylation in NFPAs, but not in other hypophyseal adenoma types [13]. In rat PAs, *Gtl2* (*Meg3*) is down-regulated (−1.95) but did not reach statistical significance.

Previous studies have compared human non-invasive and invasive/aggressive PAs with the aim to unveil the molecular mechanisms leading to an aggressive behavior and to identify markers predictive of invasiveness [12, 19, 43]. Some genes or gene families we found up-regulated in MENX adenomas had previously been identified in human aggressive-invasive PAs, e.g., *PTTG1*, *RACGAP1*, *CCNB1*, *CENPE*, *AURKB* [12]. PTTG1, the homologue of yeast securin, controls the faithful separation of sister chromatids [46], while deregulated expression of centrosome proteins (CENP) and Aurora kinases has been linked to defects in the mitotic machinery [41, 44]. Differential expression of the five genes mentioned above was observed among benign, pre-malignant and malignant tumors, with a tendency to higher expression in the more malignant ones, with *Pttg1* being expressed only in the latter group. Altogether, these findings further support our initial hypothesis that MENX-associated PAs resemble aggressive tumors [26], and suggest that these lesions may be exploited to identify novel targets for specific therapies of aggressive PAs, currently orphan of specific molecular-targeted treatment strategies. Moreover, our data further confirm the critical role of *Pttg1* in pituitary tumorigenesis across different species and genetic backgrounds.

*NUSAP1* is among the genes overexpressed in both rat and human PAs but has not so far been investigated in these tumors. We here report that 90 % of human gonadotroph adenomas express the *NUSAP1* gene at high level. Concordantly, the number of cells immunoreactive for NuSAP was higher in tumors compared with normal pituitaries. NuSAP is a microtubule-associated protein that bundles and stabilizes microtubules thereby linking chromosomes to the mitotic spindle [35]. Recently, overexpression of *NUSAP1* has been found in various human cancers by array analysis, including glioblastomas, hepatocellular carcinomas, pancreatic adenocarcinoma (reviewed in [21]). The high expression of the *NUSAP1* gene has been associated with poor prognosis in melanoma patients [3], with a malignancy-risk genetic signature in breast cancer [4] and with recurrence in prostate cancer [15]. The study on prostate cancer has validated gene up-regulation seen by array analysis with immunostaining for the NuSAP protein: tumors with Gleason grading scores ≥7 had more NuSAP-positive cells than tumors with lower scores [15]. We estimated the percentage of NuSAP-positive cells in human gonadotroph tumors (average 0.5 %, range 0.2–1 %) and found a correlation with that of Ki-67-positive cells (average 1.2 %, range 0.5–2 %). Further studies are required to determine whether NuSAP labeling index correlates with the outcome of patients with PA and whether it provides a more accurate estimate of aggressive behavior than Ki67. Noteworthy, in addition to *NUSAP1*, our meta-analysis of human PA array data has identified the up-regulation of genes encoding other proteins playing a critical role in mitosis, mitotic spindle checkpoint and dynamics, or cytokinesis such as *BUB1*, *CCNB1*, *CDC2*, *KIF4*, *KIF11, PRC1*, in gonadotroph adenomas (Supplementary Table 2). These genes, if experimentally verified to be involved in PAs, may represent novel therapeutic targets for these tumors. While classical antimitotic compounds have limited clinical applications because of severe side effects, a new generation of drugs has been developed that targets kinesins (KIF proteins) and kinases with unique function in mitosis. These antitumor agents could be tested for the treatment of gonadotroph adenomas.

We observed that SF-1 and P450scc are co-expressed in PA cells of MENX-affected animals, and both siRNA-mediated silencing of the *Nr5a1* gene and treatment with the SF-1 inhibitor IsoQ resulted in the down-regulation of *Cyp11a1* in rat primary tumor cells and in LβT2 gonadotroph cells. These results indicate that SF-1 regulates *Cyp11a1* expression in gonadotroph cells. The transcriptional regulation of *Cyp11a1* in steroidogenic tissues is complex and involves the concerted action of several tissue-specific trans-regulators such as SF-1, DAX1, TReP-132, LBP, and GATA together with transcription factors having a more widespread expression, including AP-1, Sp1, and AP-2 [16]. Further studies are needed to identify the additional factors involved in the transcriptional regulation of *Cyp11a1* in PA cells.

*CYP11A1* was up-regulated in 75 % of human gonadotropinomas and P450scc protein expression was high in these tumors, while barely detectable in normal pituitaries. P450scc has been detected in the developing mouse at embryonic day 9.5 in Rathke’s pouch and at day 18.5 in the pituitary primordium [17]. However, its potential role in pituitary development or in the adult pituitary has not been addressed. Here we show that *Cyp11a1* plays a pro-proliferative role in Y1 adrenocortical cancer cells, but also in GH3 somatotroph adenoma cells and in rat primary pituitary gonadotroph cells. To the best of our knowledge, only two studies have so far addressed the putative role of *Cyp11a1* in regulating cell proliferation or viability. The first one showed that overexpression of *CYP11A1* in tumorigenic and non-tumorigenic mammalian cell lines can either inhibit or enhance cell viability in a cell type-specific manner [7]. In cells sensitive to *CYP11A1*, gene overexpression led to decreased cell proliferation through increase of p21 expression and induction of apoptosis [7]. The authors did not investigate the molecular processes associated with *CYP11A1*-mediated increase in cell proliferation. Based on our results, when the reduction of *CYP11A1* level decreases cell proliferation (as seen in PA cells), both apoptosis and p21 expression are also involved, indicating that these are common pathways downstream of *CYP11A1* signaling. In a more recent study, a truncated isoform of P450scc was found to suppress osteoblast proliferation in physiological conditions [38]. Unlike the full length P450scc isoform, which is located in the mitochondria, this small isoform localizes to cytoplasm and nucleus, and might therefore be involved in still unidentified molecular pathways. Our studies show that *Cyp11a1* plays a pro-proliferative role in adrenocortical cancer and PA cells, thereby providing additional evidence supporting a role for this gene in tumorigenesis.

In conclusion, we here provide experimental evidence that the MENX animal model can be exploited as experimental tool to shed light into the pathomechanisms involved in human pituitary tumorigenesis. Several of the dysregulated genes here reported have never been associated with PAs, and may represent novel biomarkers for future clinical applications.

## Electronic supplementary material

Below is the link to the electronic supplementary material.

Supplementary material 1 (PPT 1545 kb)

Supplementary material 2 (DOC 259 kb)

Supplementary material 3 (XLS 144 kb)

Supplementary material 4 (PPT 105 kb)
