# Supplementary material for: Transcriptome analysis of MENX-associated rat pituitary adenomas identifies novel molecular mechanisms involved in the pathogenesis of human pituitary gonadotroph adenomas
Source: Acta Neuropathol. 2013 Jun 12;126(1):137–50. doi: 10.1007/s00401-013-1132-7 (PMC3690182; doi:10.1007/s00401-013-1132-7)
Supplement: Supplementary file 1 — Supplementary material 1 (PPT 1545 kb) [file 401_2013_1132_MOESM1_ESM.ppt]

## Slide 1
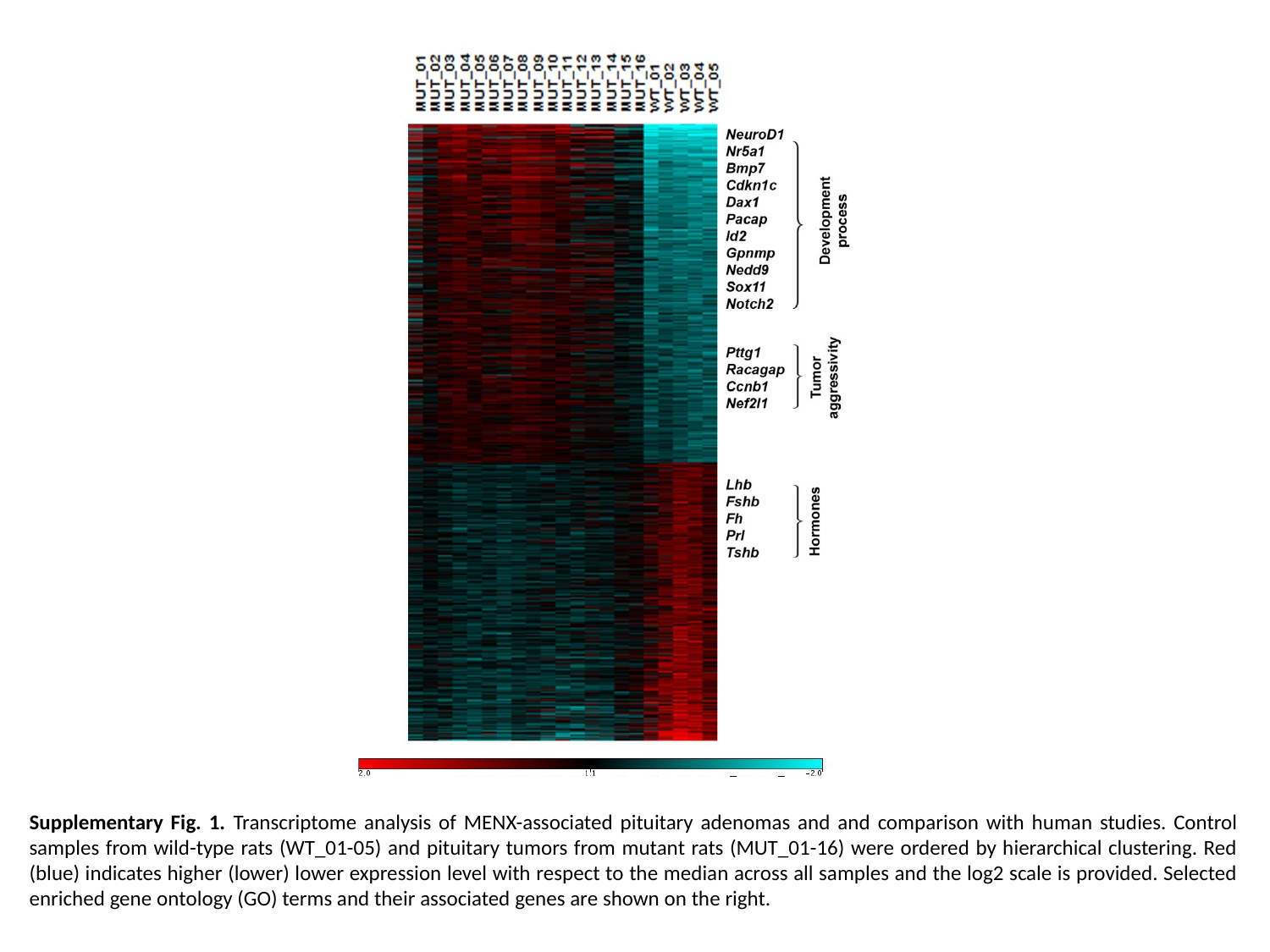

Supplementary Fig. 1. Transcriptome analysis of MENX-associated pituitary adenomas and and comparison with human studies. Control samples from wild-type rats (WT_01-05) and pituitary tumors from mutant rats (MUT_01-16) were ordered by hierarchical clustering. Red (blue) indicates higher (lower) lower expression level with respect to the median across all samples and the log2 scale is provided. Selected enriched gene ontology (GO) terms and their associated genes are shown on the right.

## Slide 2
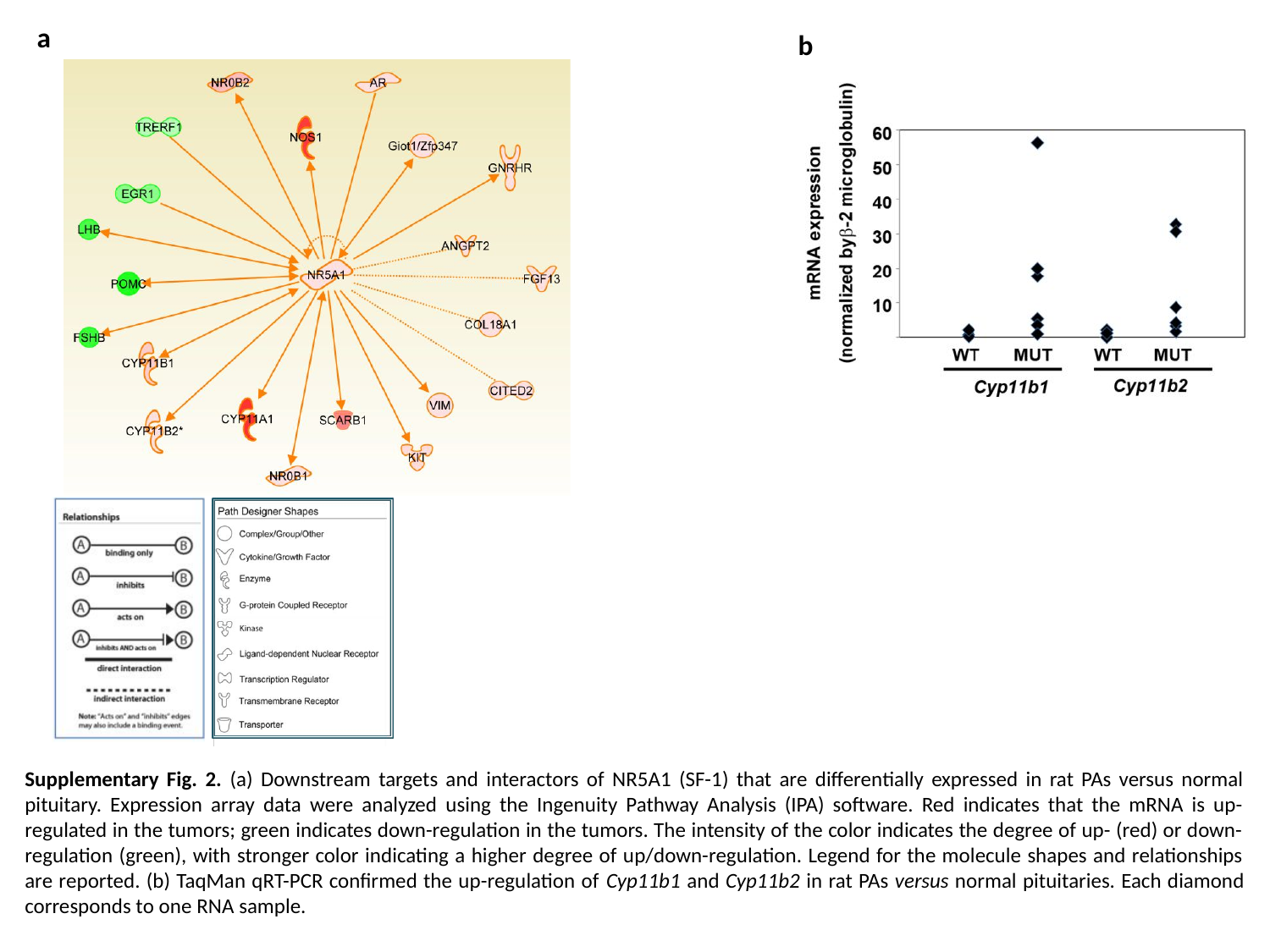

a
b
Supplementary Fig. 2. (a) Downstream targets and interactors of NR5A1 (SF-1) that are differentially expressed in rat PAs versus normal pituitary. Expression array data were analyzed using the Ingenuity Pathway Analysis (IPA) software. Red indicates that the mRNA is up-regulated in the tumors; green indicates down-regulation in the tumors. The intensity of the color indicates the degree of up- (red) or down-regulation (green), with stronger color indicating a higher degree of up/down-regulation. Legend for the molecule shapes and relationships are reported. (b) TaqMan qRT-PCR confirmed the up-regulation of Cyp11b1 and Cyp11b2 in rat PAs versus normal pituitaries. Each diamond corresponds to one RNA sample.

## Slide 3
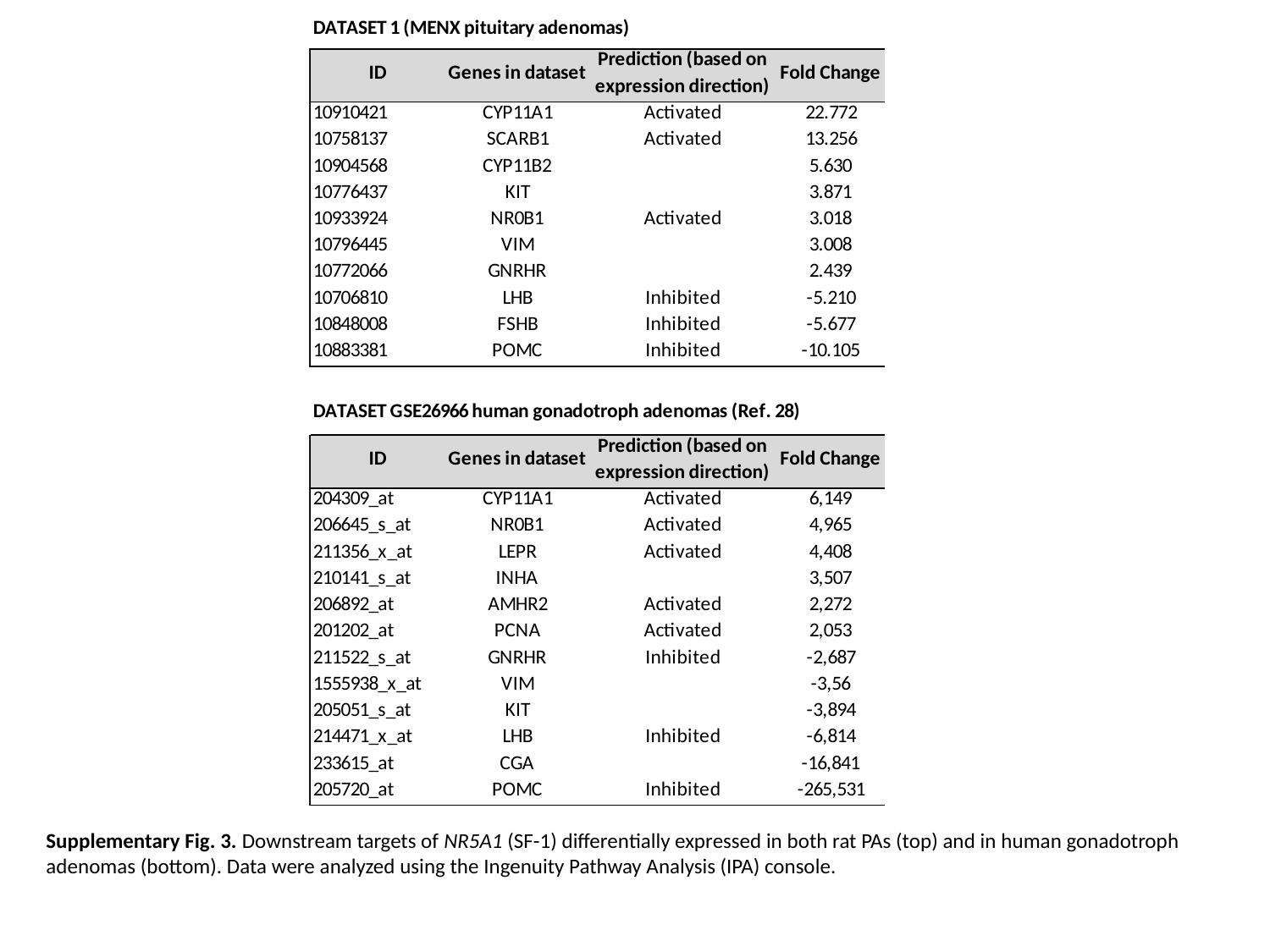

Supplementary Fig. 3. Downstream targets of NR5A1 (SF-1) differentially expressed in both rat PAs (top) and in human gonadotroph adenomas (bottom). Data were analyzed using the Ingenuity Pathway Analysis (IPA) console.

## Slide 4
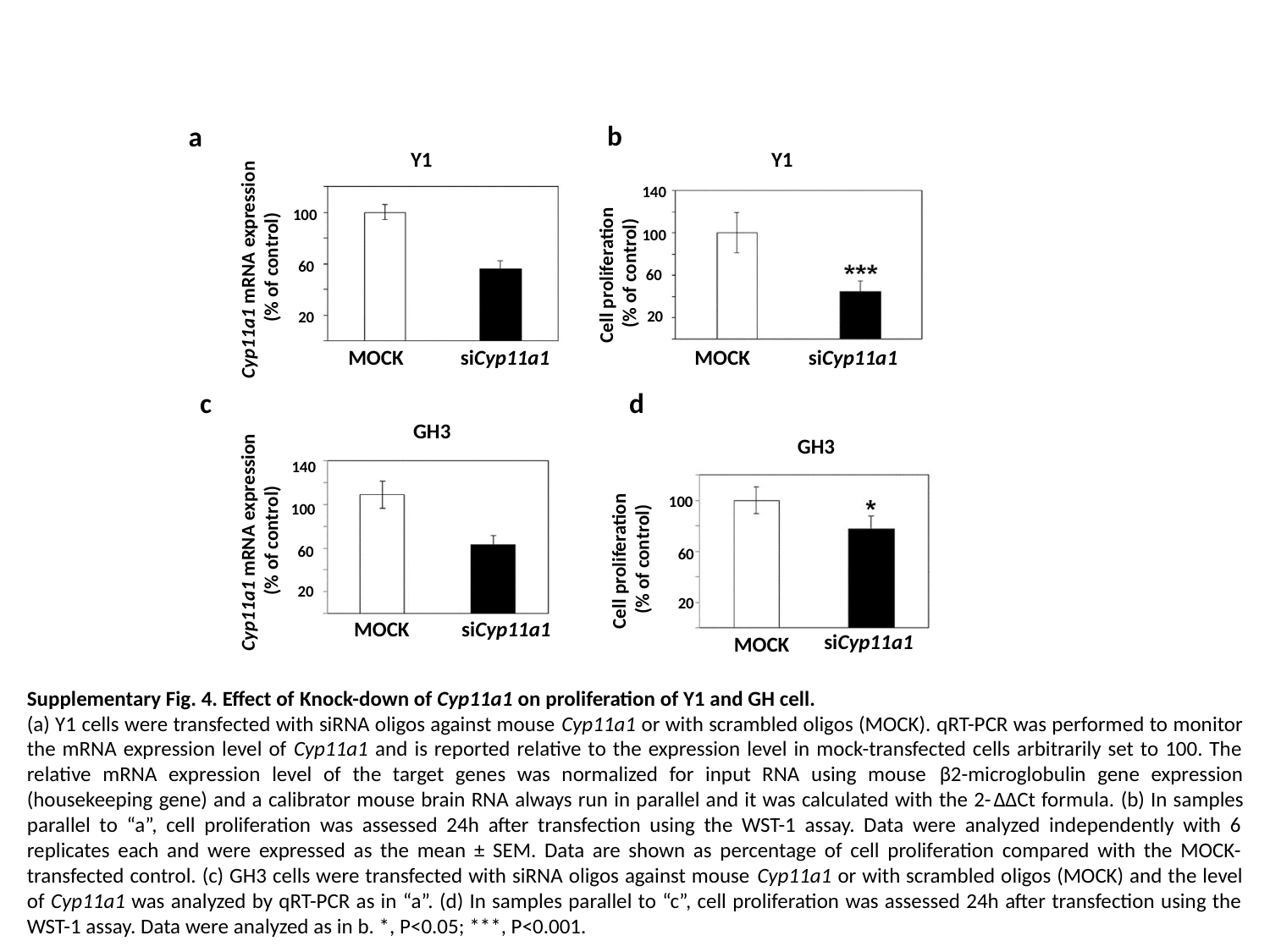

b
a
Y1
Y1
140
100
100
Cyp11a1 mRNA expression
(% of control)
Cell proliferation
(% of control)
60
60
20
20
MOCK
siCyp11a1
MOCK
siCyp11a1
c
d
GH3
GH3
140
100
100
Cyp11a1 mRNA expression
(% of control)
Cell proliferation
(% of control)
60
60
20
20
MOCK
siCyp11a1
siCyp11a1
MOCK
Supplementary Fig. 4. Effect of Knock-down of Cyp11a1 on proliferation of Y1 and GH cell.
(a) Y1 cells were transfected with siRNA oligos against mouse Cyp11a1 or with scrambled oligos (MOCK). qRT-PCR was performed to monitor the mRNA expression level of Cyp11a1 and is reported relative to the expression level in mock-transfected cells arbitrarily set to 100. The relative mRNA expression level of the target genes was normalized for input RNA using mouse β2-microglobulin gene expression (housekeeping gene) and a calibrator mouse brain RNA always run in parallel and it was calculated with the 2-ΔΔCt formula. (b) In samples parallel to “a”, cell proliferation was assessed 24h after transfection using the WST-1 assay. Data were analyzed independently with 6 replicates each and were expressed as the mean ± SEM. Data are shown as percentage of cell proliferation compared with the MOCK-transfected control. (c) GH3 cells were transfected with siRNA oligos against mouse Cyp11a1 or with scrambled oligos (MOCK) and the level of Cyp11a1 was analyzed by qRT-PCR as in “a”. (d) In samples parallel to “c”, cell proliferation was assessed 24h after transfection using the WST-1 assay. Data were analyzed as in b. *, P<0.05; ***, P<0.001.

## Slide 5
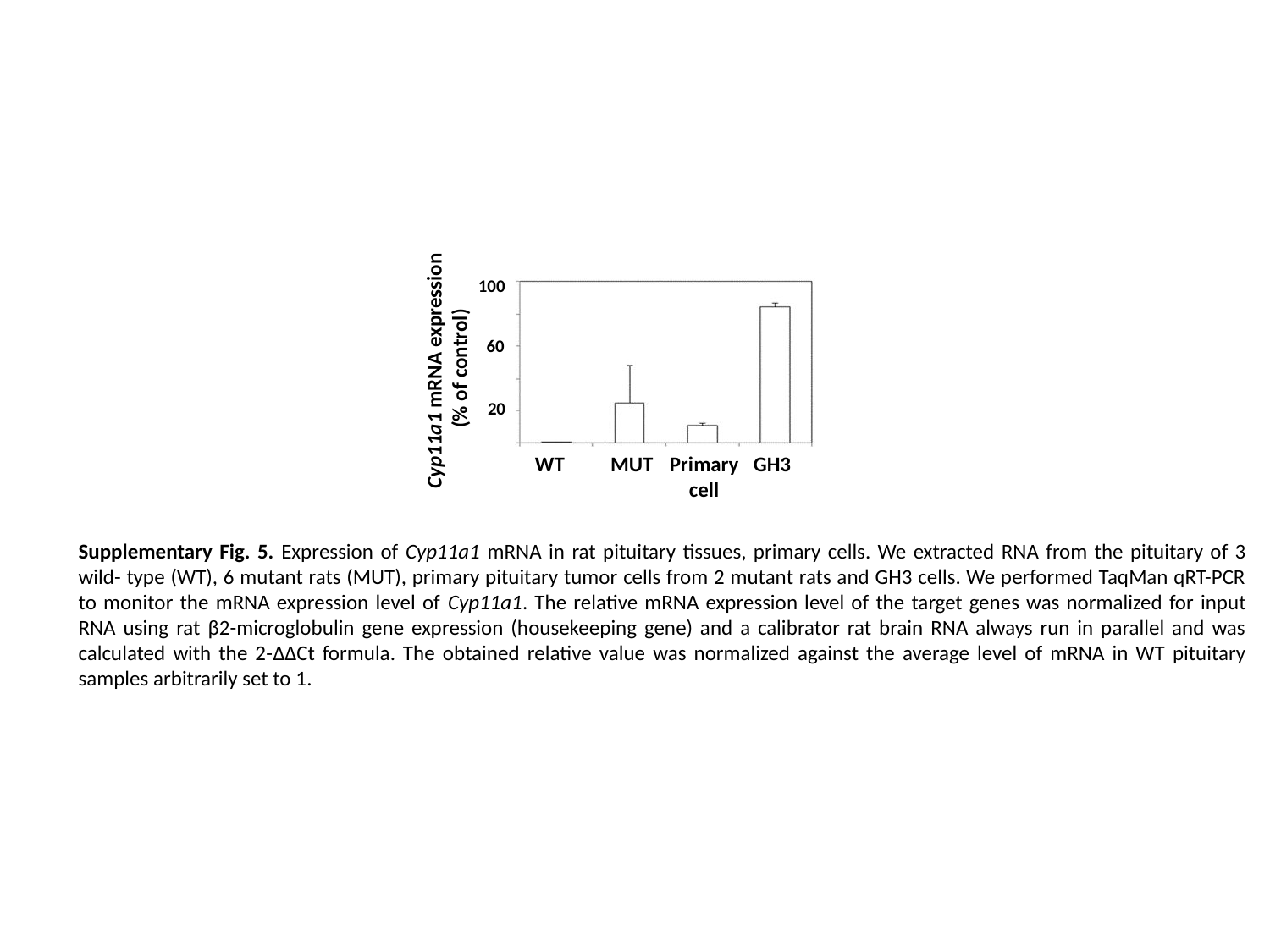

100
60
20
WT
MUT
Primary cell
GH3
Cyp11a1 mRNA expression
(% of control)
Supplementary Fig. 5. Expression of Cyp11a1 mRNA in rat pituitary tissues, primary cells. We extracted RNA from the pituitary of 3 wild- type (WT), 6 mutant rats (MUT), primary pituitary tumor cells from 2 mutant rats and GH3 cells. We performed TaqMan qRT-PCR to monitor the mRNA expression level of Cyp11a1. The relative mRNA expression level of the target genes was normalized for input RNA using rat β2-microglobulin gene expression (housekeeping gene) and a calibrator rat brain RNA always run in parallel and was calculated with the 2-ΔΔCt formula. The obtained relative value was normalized against the average level of mRNA in WT pituitary samples arbitrarily set to 1.

## Slide 6
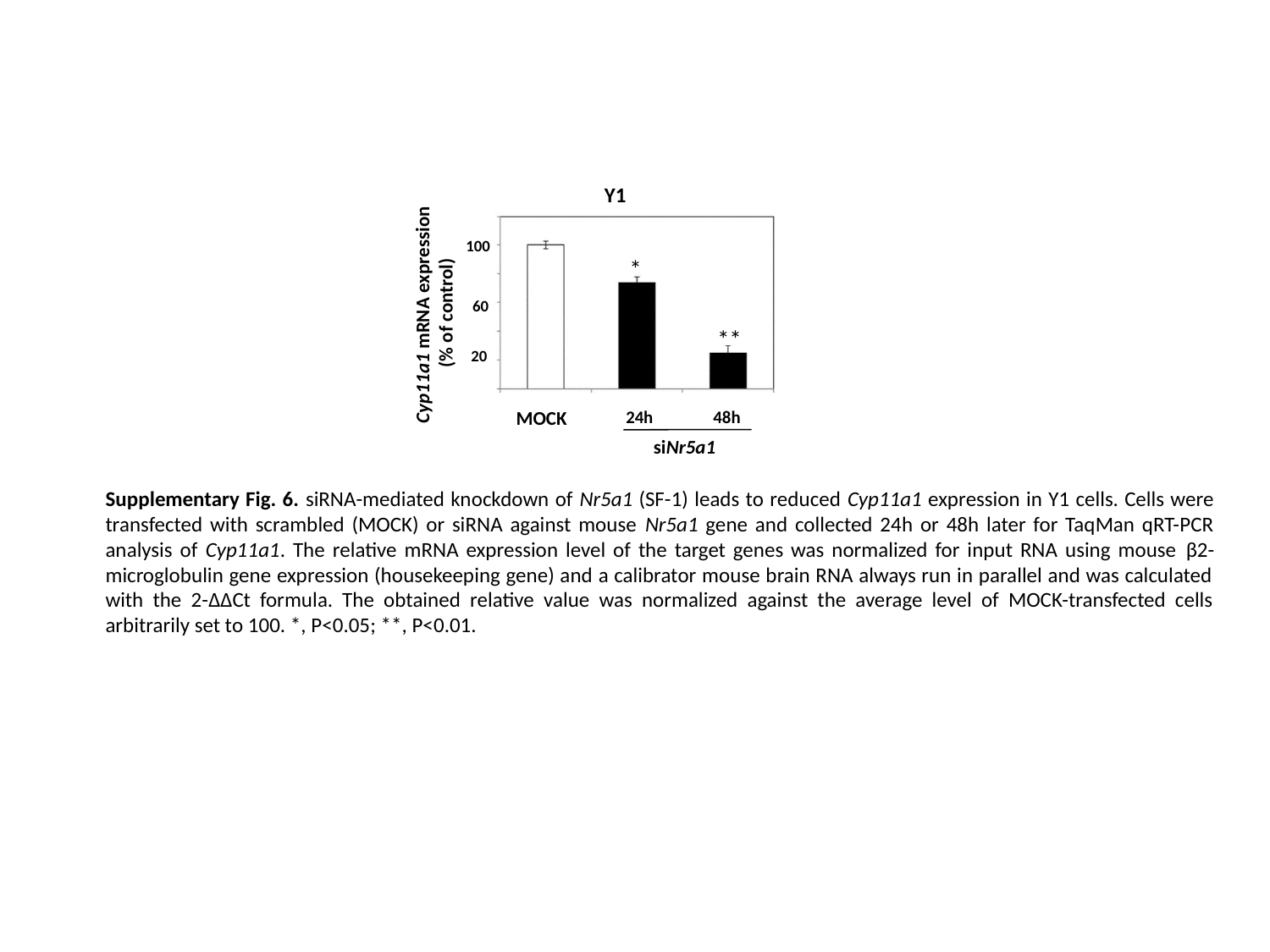

Y1
100
60
20
24h
48h
MOCK
siNr5a1
Cyp11a1 mRNA expression
(% of control)
*
**
Supplementary Fig. 6. siRNA-mediated knockdown of Nr5a1 (SF-1) leads to reduced Cyp11a1 expression in Y1 cells. Cells were transfected with scrambled (MOCK) or siRNA against mouse Nr5a1 gene and collected 24h or 48h later for TaqMan qRT-PCR analysis of Cyp11a1. The relative mRNA expression level of the target genes was normalized for input RNA using mouse β2-microglobulin gene expression (housekeeping gene) and a calibrator mouse brain RNA always run in parallel and was calculated with the 2-ΔΔCt formula. The obtained relative value was normalized against the average level of MOCK-transfected cells arbitrarily set to 100. *, P<0.05; **, P<0.01.
