# Supplementary material for: Transcriptome analysis of MENX-associated rat pituitary adenomas identifies novel molecular mechanisms involved in the pathogenesis of human pituitary gonadotroph adenomas
Source: Acta Neuropathol. 2013 Jun 12;126(1):137–50. doi: 10.1007/s00401-013-1132-7 (PMC3690182; doi:10.1007/s00401-013-1132-7)
Supplement: Supplementary file 2 — Supplementary material 2 (DOC 259 kb) [file 401_2013_1132_MOESM2_ESM.doc]

**Supplementary Table 2.** Nonredundant list of genes concordantly dysregulated in rat pituitary tumors from MENX rats (887 probe sets) and in datasets based on Michaelis et al. (28), Morris et al. (32) and Moreno et al. (31). In bold are indicated the *Cyp11a1* and *Nusap1* genes that we selected for further studies.

| **Probe set ID** | **Gene symbol** | **Gene description** | **Rat Entrez gene ID** | **Fold change in  MENX rats  (Tumor/WT)** | **Fold change (Tumor/WT),  Michaelis et al.  (28)** |
| --- | --- | --- | --- | --- | --- |
| **10910421** | **Cyp11a1** | **cytochrome P450, family 11, subfamily a, polypeptide 1** | **29680** | **22,8** | **6,1** |
| 10875616 | Necab1 | N-terminal EF-hand calcium binding protein 1 | 64169 | 17,5 | 13,9 |
| 10925783 | Man2a1 | mannosidase, alpha, class 2A, member 1 | 25478 | 12,5 | 2,2 |
| 10846685 | Neurod1 | neurogenic differentiation 1 | 29458 | 11,0 | 5,7 |
| 10781146 | Pbk | PDZ binding kinase | 290326 | 10,2 | 3,1 |
| 10882525 | Tmem178 | transmembrane protein 178 | 362691 | 7,5 | 4,8 |
| 10822558 | Ect2 | epithelial cell transforming sequence 2 oncogene | 361921 | 6,9 | 2,1 |
| 10764495 | B3galt2 | UDP-Gal:betaGlcNAc beta 1,3-galactosyltransferase,  polypeptide 2 | 686081 | 6,2 | 2,6 |
| 10821016 | Ccnb1 | cyclin B1 | 25203 | 5,6 | 3,2 |
| 10764032 | Etnk2 | ethanolamine kinase 2 | 360843 | 4,9 | 3,8 |
| 10882475 | Fam82a1 | family with sequence similarity 82, member A1 | 313840 | 4,8 | 2,2 |
| 10840942 | Ttll9 | tubulin tyrosine ligase-like family, member 9 | 311548 | 4,8 | 2,4 |
| 10837175 | Ssfa2 | sperm specific antigen 2 | 311146 | 4,3 | 3,8 |
| **10838843** | **Nusap1** | **nucleolar and spindle associated protein 1** | **311336** | **4,2** | **8,4** |
| 10772448 | Cnga1 | cyclic nucleotide gated channel alpha 1 | 85259 | 4,1 | 6,2 |
| 10789819 | Cacna2d3 | calcium channel, voltage-dependent, alpha2 | 306243 | 3,9 | 4,7 |
| 10708214 | Prc1 | protein regulator of cytokinesis 1 | 308761 | 3,7 | 3,1 |
| 10751091 | Cd200 | Cd200 molecule | 24560 | 3,5 | 2,3 |
| 10820523 | Pde8b | phosphodiesterase 8B | 309962 | 3,5 | 3,2 |
| 10867821 | Ggh | gamma-glutamyl hydrolase | 25455 | 3,4 | 2,7 |
| 10802795 | St8sia5 | ST8 alpha-N-acetyl-neuraminide alpha- 2,8-sialyltransferase 5 | 364901 | 3,3 | 3,1 |
| 10934239 | Kif4 | kinesin family member 4 | 84393 | 3,3 | 8,4 |
| 10868693 | Frmpd1 | FERM and PDZ domain containing 1 | 313244 | 3,3 | 3,4 |
| 10776582 | Slc10a4 | Solute carrier family 10 member 4 | 305309 | 3,2 | 12,2 |
| 10907178 | Racgap1 | Rac GTPase-activating protein 1 | 315298 | 3,2 | 2,6 |
| 10857969 | Syn2 | synapsin II | 29179 | 3,1 | 3,4 |
| 10794609 | Elovl2 | elongation of very long chain fatty acids (FEN1 | 498728 | 3,1 | 5,0 |
| 10933924 | Nr0b1 | nuclear receptor subfamily 0, group B, member 1 | 58850 | 3,0 | 5,0 |
| 10770728 | Dtl | denticleless homolog (Drosophila) | 305073 | 2,9 | 2,7 |
| 10846652 | Zfp385b | zinc finger protein 385B | 311137 | 2,8 | 3,1 |
| 10746804 | Plxdc1 | plexin domain containing 1 | 303505 | 2,8 | 2,2 |
| 10752679 | Cadm2 | cell adhesion molecule 2 | 360687 | 2,7 | 3,9 |
| 10823057 | Slc7a11 | Solute carrier family 7 member 11 | 310392 | 2,6 | 4,8 |
| 10870369 | Sgip1 | SH3-domain GRB2-like (endophilin) interacting protein 1 | 313413 | 2,5 | 3,9 |
| 10910406 | Sema7a | Semaphorin 7A, GPI membrane anchor  (John Milton Hagen blood group) | 315711 | 2,5 | 3,6 |
| 10934118 | Ar | androgen receptor | 24208 | 2,5 | 3,9 |
| 10742128 | Hmmr | hyaluronan mediated motility receptor (RHAMM) | 25460 | 2,5 | 2,2 |
| 10870342 | Pde4b | phosphodiesterase 4B, cAMP specific | 24626 | 2,5 | 3,9 |
| 10893899 | Gamt | guanidinoacetate N-methyltransferase | 25257 | 2,4 | 2,4 |
| 10900511 | Reep6 | receptor accessory protein 6 | 362835 | 2,4 | 3,6 |
| 10715078 | Kif11 | kinesin family member 11 | 171304 | 2,4 | 3,1 |
| 10753839 | RGD1310335 | similar to RIKEN cDNA C330027C09 | 360711 | 2,3 | 2,3 |
| 10838647 | Bub1b | budding uninhibited by benzimidazoles 1 homolog,  beta (S. cerevisiae) | 171576 | 2,3 | 3,2 |
| 10775484 | Arhgap24 | Rho GTPase activating protein 24 | 305156 | 2,3 | 3,1 |
| 10759009 | Cabp1 | calcium binding protein 1 | 171051 | 2,2 | 4,3 |
| 10778028 | Tcn2 | transcobalamin 2 | 64365 | 2,2 | 3,5 |
| 10837195 | Ppp1r1c | protein phosphatase 1, regulatory (inhibitor) subunit 1C | 499818 | 2,2 | 2,9 |
| 10717170 | Epb4.1l2 | erythrocyte membrane protein band 4.1-like 2 | 309557 | 2,2 | 2,3 |
| 10926740 | Tnfrsf21 | tumor necrosis factor receptor superfamily, member 21 | 316256 | 2,2 | 3,7 |
| 10704960 | Cadm4 | cell adhesion molecule 4 | 365216 | 2,1 | 2,2 |
| 10868871 | Galnt12 | Polypeptide GalNAc transferase 12 | 313233 | 2,1 | 20,0 |
| 10708065 | LOC683179 | similar to CG13745-PA | 683179 | 2,0 | 2,8 |
| 10935589 | Htatsf1 | HIV-1 Tat specific factor 1 | 317612 | -2,0 | -3,0 |
| 10860801 | RGD1563091 | similar to OEF2 | 500011 | -2,0 | -2,1 |
| 10848393 | Rasgrp1 | RAS guanyl releasing protein 1 (calcium and DAG-regulated) | 29434 | -2,0 | -2,1 |
| 10936377 | Dock11 | dedicator of cytokinesis 11 | 313438 | -2,0 | -10,6 |
| 10901306 | Rfx4 | regulatory factor X, 4 (influences HLA class II expression) | 500818 | -2,0 | -5,9 |
| 10926162 | Kcnh8 | potassium voltage-gated channel,  subfamily H (eag-related), member 8 | 246325 | -2,1 | -2,2 |
| 10749826 | Pou1f1 | POU class 1 homeobox 1 | 25517 | -2,1 | -66,0 |
| 10782187 | Itgbl1 | integrin, beta-like 1 | 498564 | -2,1 | -5,2 |
| 10793475 | Cdc14b | CDC14 cell division cycle 14 homolog B (S. cerevisiae) | 361195 | -2,1 | -3,0 |
| 10717118 | Ptprk | protein tyrosine phosphatase, receptor type, K, extracellular region | 360302 | -2,1 | -4,4 |
| 10751404 | Slc15a2 | solute carrier family 15 member 2 | 60577 | -2,1 | -2,4 |
| 10853819 | Met | met proto-oncogene | 24553 | -2,1 | -5,9 |
| 10777137 | Slit2 | slit homolog 2 (Drosophila) | 360272 | -2,1 | -5,3 |
| 10779464 | Fam107a | family with sequence similarity 107, member A | 361018 | -2,1 | -3,0 |
| 10859392 | Mgst1 | microsomal glutathione S-transferase 1 | 171341 | -2,1 | -7,0 |
| 10821824 | Slc1a3 | Solute carrier family 1 member 3 | 29483 | -2,2 | -2,5 |
| 10855707 | Ghrhr | growth hormone releasing hormone receptor | 25321 | -2,2 | -61,7 |
| 10817552 | Txnip | thioredoxin interacting protein | 117514 | -2,2 | -3,2 |
| 10812689 | Enc1 | ectodermal-neural cortex 1 | 294674 | -2,2 | -2,5 |
| 10786100 | Fgf14 | fibroblast growth factor 14 | 63851 | -2,2 | -3,5 |
| 10798390 | Fam65b | family with sequence similarity 65, member B | 306934 | -2,2 | -2,6 |
| 10874903 | Pi15 | peptidase inhibitor 15 | 301489 | -2,2 | -2,5 |
| 10886031 | Fos | FBJ osteosarcoma oncogene | 314322 | -2,2 | -8,5 |
| 10784120 | Gjb6 | gap junction protein, beta 6 | 84403 | -2,2 | -2,9 |
| 10822757 | Ccdc39 | coiled-coil domain containing 39 | 310315 | -2,2 | -2,5 |
| 10726085 | Prss8 | protease, serine, 8 | 192107 | -2,2 | -2,0 |
| 10758663 | Aldh2 | aldehyde dehydrogenase 2 family (mitochondrial) | 29539 | -2,2 | -2,5 |
| 10848176 | Ryr3 | ryanodine receptor 3 | 170546 | -2,2 | -2,8 |
| 10877755 | Nfib | nuclear factor I | 29227 | -2,2 | -4,3 |
| 10767763 | Prelp | proline/arginine-rich end leucine-rich repeat protein | 84400 | -2,2 | -6,8 |
| 10726255 | Cpxm2 | carboxypeptidase X (M14 family), member 2 | 293566 | -2,3 | -3,9 |
| 10860878 | Pon3 | paraoxonase 3 | 312086 | -2,3 | -2,3 |
| 10817711 | Notch2 | Notch homolog 2 (Drosophila) | 29492 | -2,3 | -2,9 |
| 10781829 | Klf5 | Kruppel-like factor 5 | 84410 | -2,4 | -3,1 |
| 10909590 | Mpzl2 | myelin protein zero-like 2 | 300679 | -2,4 | -3,5 |
| 10824530 | Il6ra | interleukin 6 receptor, alpha | 24499 | -2,4 | -12,6 |
| 10891765 | Tc2n | tandem C2 domains, nuclear | 500707 | -2,5 | -3,3 |
| 10752576 | Pkp2 | plakophilin 2 | 287925 | -2,5 | -2,5 |
| 10868940 | Nr4a3 | nuclear receptor subfamily 4, group A, member 3 | 58853 | -2,6 | -9,3 |
| 10707953 | Akap13 | A kinase (PRKA) anchor protein 13 | 293024 | -2,7 | -2,8 |
| 10739986 | Rnf213 | ring finger protein 213 | 303735 | -2,7 | -2,5 |
| 10729970 | Rbp4 | retinol binding protein 4, plasma | 25703 | -2,7 | -2,9 |
| 10753269 | Kcnj6 | potassium inwardly-rectifying channel, subfamily J, member 6 | 25743 | -2,8 | -13,5 |
| 10870261 | Ak3l1 | adenylate kinase 3-like 1 | 29223 | -2,8 | -3,0 |
| 10939764 | Gpc3 | glypican 3 | 25236 | -2,9 | -6,8 |
| 10764460 | Kcnt2 | potassium channel, subfamily T, member 2 | 304827 | -3,0 | -3,2 |
| 10935390 | RGD1565967 | similar to hypothetical protein FLJ30058 | 302819 | -3,1 | -28,3 |
| 10931271 | Ces3 | carboxylesterase 3 | 113902 | -3,1 | -2,2 |
| 10843460 | Ptgds | prostaglandin D2 synthase (brain) | 25526 | -3,3 | -12,6 |
| 10800919 | Egr1 | early growth response 1 | 24330 | -3,3 | -5,3 |
| 10734082 | Myo15 | myosin XV | 501699 | -3,3 | -4,7 |
| 10845384 | Nr4a2 | nuclear receptor subfamily 4, group A, member 2 | 54278 | -3,4 | -38,9 |
| 10886806 | Dlk1 | delta-like 1 homolog (Drosophila) | 114587 | -3,4 | -601,5 |
| 10903529 | Angpt1 | angiopoietin 1 | 89807 | -3,4 | -9,7 |
| 10714323 | Aldh1a1 | aldehyde dehydrogenase 1 family, member A1 | 24188 | -3,6 | -3,3 |
| 10836271 | Kcnj3 | potassium inwardly-rectifying channel, subfamily J, member 3 | 50599 | -3,7 | -2,3 |
| 10919637 | Tf | transferrin | 24825 | -3,8 | -3,6 |
| 10776631 | Gabrb1 | gamma-aminobutyric acid (GABA) A receptor, beta 1 | 25450 | -4,9 | -3,6 |
| 10706810 | Lhb | luteinizing hormone beta | 25329 | -5,2 | -6,8 |
| 10825521 | Tshb | thyroid stimulating hormone, beta | 25653 | -5,6 | -202,6 |
| 10772522 | Gabra4 | gamma-aminobutyric acid (GABA) A receptor, alpha 4 | 140675 | -8,1 | -7,5 |
| 10883381 | Pomc | proopiomelanocortin | 24664 | -10,1 | -265,5 |

| **Probe set ID** | **Gene symbol** | **Gene description** | **Rat Entrez gene ID** | **Fold change in MENX rats (Tumor/WT)** | **Fold change (Tumor/WT), Morris et al. (32)** |
| --- | --- | --- | --- | --- | --- |
| 10931930 | Fam70a | family with sequence similarity 70, member A | 313453 | 2,8 | 11,8 |
| 10939919 | Fgf13 | fibroblast growth factor 13 | 84488 | 3,3 | 5,0 |
| 10857969 | Syn2 | synapsin II | 29179 | 3,1 | 3,0 |
| 10772169 | Igfbp7 | insulin-like growth factor binding protein 7 | 289560 | 2,8 | 2,9 |
| **10838843** | **Nusap1** | **nucleolar and spindle associated protein 1** | **311336** | **4,2** | **2,7** |
| 10805186 | Me2 | malic enzyme 2, NAD(+)-dependent, mitochondrial | 307270 | 4,1 | 2,7 |
| 10829761 | Cdc2 | cell division cycle 2, G1 to S and G2 to M | 54237 | 2,4 | 2,6 |
| 10784125 | Cryl1 | crystallin, lambda 1 | 290277 | 2,4 | 2,5 |
| 10782919 | Gch1 | GTP cyclohydrolase 1 | 29244 | 3,3 | 2,4 |
| 10758896 | Pebp1 | phosphatidylethanolamine binding protein 1 | 29542 | 3,3 | 2,4 |
| 10853453 | Pftk1 | PFTAIRE protein kinase 1 | 362316 | 2,8 | 2,4 |
| 10764661 | Fam129a | family with sequence similarity 129, member A | 63912 | 3,5 | 2,3 |
| 10798048 | Cdyl | chromodomain protein, Y-like | 361237 | 2,4 | 2,3 |
| 10751048 | Tagln3 | transgelin 3 | 63837 | 8,0 | 2,2 |
| 10809942 | Dhps | deoxyhypusine synthase | 288923 | 2,0 | 2,2 |
| 10844666 | Stom | stomatin | 296655 | 2,1 | 2,1 |
| 10770143 | Fh1 | fumarate hydratase 1 | 24368 | 2,4 | 2,1 |
| 10903736 | Enpp2 | ectonucleotide pyrophosphatase | 84050 | 2,7 | 2,0 |
| 10926930 | Mcm3 | minichromosome maintenance complex component 3 | 316273 | 2,1 | 2,0 |
| 10867461 | Lyn | v-yes-1 Yamaguchi sarcoma viral related oncogene homolog | 81515 | 2,4 | 2,0 |
| 10921772 | Vegfa | vascular endothelial growth factor A | 83785 | 2,4 | 2,0 |
| 10746804 | Plxdc1 | plexin domain containing 1 | 303505 | 2,8 | 1,9 |
| 10876052 | Bag1 | BCL2-associated athanogene | 297994 | 2,3 | 1,8 |
| 10909446 | Mcam | melanoma cell adhesion molecule | 78967 | 2,1 | 1,8 |
| 10855707 | Ghrhr | growth hormone releasing hormone receptor | 25321 | -2,2 | -2,2 |
| 10734082 | Myo15 | myosin XV | 501699 | -3,3 | -2,4 |
| 10845384 | Nr4a2 | nuclear receptor subfamily 4, group A, member 2 | 54278 | -3,4 | -2,4 |
| 10919637 | Tf | transferrin | 24825 | -3,8 | -3,2 |
| 10717118 | Ptprk | protein tyrosine phosphatase, receptor type, K | 360302 | -2,1 | -3,4 |
| 10824530 | Il6ra | interleukin 6 receptor, alpha | 24499 | -2,4 | -4,2 |
| 10903529 | Angpt1 | angiopoietin 1 | 89807 | -3,4 | -4,6 |
| 10769492 | Tbx19 | T-box 19 | 304935 | -4,1 | -6,1 |
| 10800919 | Egr1 | early growth response 1 | 24330 | -3,3 | -7,6 |
| 10749826 | Pou1f1 | POU class 1 homeobox 1 | 25517 | -2,1 | -11,9 |
| 10886031 | Fos | FBJ osteosarcoma oncogene | 314322 | -2,2 | -18,3 |
| 10860878 | Pon3 | paraoxonase 3 | 312086 | -2,3 | -23,2 |

| **Probe set ID** | **Gene symbol** | **Gene description** | **Rat Entrez gene ID** | **Fold change MENX rats (Tumor/WT)** | **Fold change (Tumor/WT) Moreno et al. (31)** |
| --- | --- | --- | --- | --- | --- |
| **10910421** | **Cyp11a1** | **cytochrome P450, family 11, subfamily a, polypeptide 1** | **29680** | **22,77** | **4,1** |
| 10846685 | Neurod1 | neurogenic differentiation 1 | 29458 | 10,95 | 3,8 |
| 10759009 | Cabp1 | calcium binding protein 1 | 171051 | 2,22 | 2,7 |
| 10751091 | Cd200 | Cd200 molecule | 24560 | 3,53 | 2,2 |
| 10753269 | Kcnj6 | potassium inwardly-rectifying channel,  subfamily J, member 6 | 25743 | -2,78 | -2,6 |
| 10758663 | Aldh2 | aldehyde dehydrogenase 2 family (mitochondrial) | 29539 | -2,23 | -3,2 |
| 10714323 | Aldh1a1 | aldehyde dehydrogenase 1 family, member A1 | 24188 | -3,58 | -4,8 |
| 10935589 | Htatsf1 | HIV-1 Tat specific factor 1 | 317612 | -2,01 | -5,0 |
| 10903529 | Angpt1 | angiopoietin 1 | 89807 | -3,44 | -5,3 |
| 10845384 | Nr4a2 | nuclear receptor subfamily 4, group A, member 2 | 54278 | -3,39 | -10,6 |
| 10860878 | Pon3 | paraoxonase 3 | 312086 | -2,26 | -12,5 |
| 10706810 | Lhb | luteinizing hormone beta | 25329 | -5,21 | -13,0 |
| 10717118 | Ptprk | protein tyrosine phosphatase, receptor type,  K, extracellular region | 360302 | -2,11 | -13,8 |
| 10886031 | Fos | FBJ osteosarcoma oncogene | 314322 | -2,20 | -18,5 |
| 10886806 | Dlk1 | delta-like 1 homolog (Drosophila) | 114587 | -3,40 | -917,4 |
| 10883381 | Pomc | proopiomelanocortin | 24664 | -10,11 | -1666,7 |
