# Supplementary material for: Transcriptome analysis of MENX-associated rat pituitary adenomas identifies novel molecular mechanisms involved in the pathogenesis of human pituitary gonadotroph adenomas
Source: Acta Neuropathol. 2013 Jun 12;126(1):137–50. doi: 10.1007/s00401-013-1132-7 (PMC3690182; doi:10.1007/s00401-013-1132-7)
Supplement: Supplementary file 4 — Supplementary material 4 (PPT 105 kb) [file 401_2013_1132_MOESM4_ESM.ppt]

## Slide 1
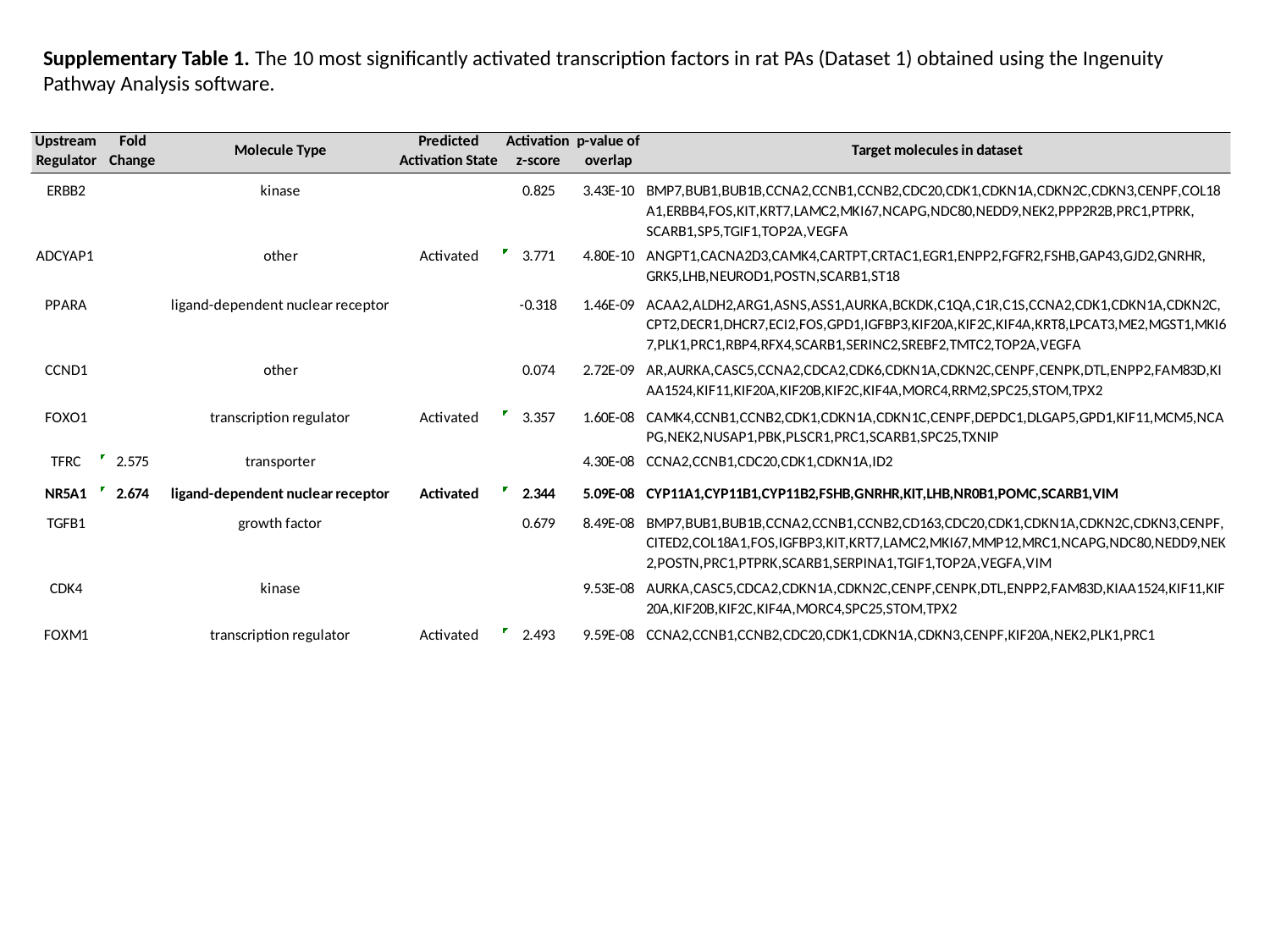

Supplementary Table 1. The 10 most significantly activated transcription factors in rat PAs (Dataset 1) obtained using the Ingenuity Pathway Analysis software.
